# Grass xylan structural variation suggests functional specialization and distinctive interaction with cellulose and lignin

**DOI:** 10.1111/tpj.16096

**Published:** 2023-01-19

**Authors:** Theodora Tryfona, Matthieu Bourdon, Rita Delgado Marques, Marta Busse‐Wicher, Francisco Vilaplana, Katherine Stott, Paul Dupree

**Affiliations:** ^1^ Department of Biochemistry, School of Biological Sciences University of Cambridge Cambridge CB2 1QW UK; ^2^ Sainsbury Laboratory University of Cambridge Cambridge CB2 1LR UK; ^3^ Department of Chemistry, School of Engineering Sciences in Chemistry, Biotechnology and Health KTH Royal Institute of Technology Stockholm SE‐10691 Sweden

**Keywords:** *Miscanthus sinensis*, *Brachypodium distachyon*, grass xylan, polysaccharide substitution patterns, grass cell wall, arabinosylation, glucuronidation, xylan–lignin interactions, xylan–cellulose interactions, cell wall molecular architecture

## Abstract

Xylan is the most abundant non‐cellulosic polysaccharide in grass cell walls, and it has important structural roles. The name glucuronoarabinoxylan (GAX) is used to describe this variable hemicellulose. It has a linear backbone of β‐1,4‐xylose (Xyl) residues that may be substituted with α‐1,2‐linked (4‐*O*‐methyl)‐glucuronic acid (GlcA), α‐1,3‐linked arabinofuranose (Ara*f*), and sometimes acetylation at the *O*‐2 and/or *O*‐3 positions. The role of these substitutions remains unclear, although there is increasing evidence that they affect the way xylan interacts with other cell wall components, particularly cellulose and lignin. Here, we used substitution‐dependent endo‐xylanase enzymes to investigate the variability of xylan substitution in grass culm cell walls. We show that there are at least three different types of xylan: (i) an arabinoxylan with evenly distributed Ara*f* substitutions without GlcA (AXe); (ii) a glucuronoarabinoxylan with clustered GlcA modifications (GAXc); and (iii) a highly substituted glucuronoarabinoxylan (hsGAX). Immunolocalization of AXe and GAXc in *Brachypodium distachyon* culms revealed that these xylan types are not restricted to a few cell types but are instead widely detected in *Brachypodium* cell walls. We hypothesize that there are functionally specialized xylan types within the grass cell wall. The even substitutions of AXe may permit folding and binding on the surface of cellulose fibrils, whereas the more complex substitutions of the other xylans may support a role in the matrix and interaction with other cell wall components.

## Significance Statement

Xylan substitutions and their arrangement can influence xylan interaction with cellulose fibrils or lignin; however, the importance in grass cell walls is poorly understood. A deeper understanding of the nature of the xylan–cellulose and xylan–lignin interactions will allow scientists to develop cell wall models, predict wall properties and identify molecular targets for bioengineering, enabling the development of better approaches to use plant biomass to produce sustainable fuels and biomaterials.

## INTRODUCTION

Plant cell walls are intricate polysaccharide‐based structures. There are two widespread and distinct developmental types of plant cell walls that differ in composition and molecular cross‐linking. Primary cell walls are highly hydrated structures, having a plastic architecture enabling the cells to expand and grow. On the other hand, secondary cell walls are less hydrated, form after the deposition of the primary cell wall, once cells cease to expand, and are rigid structures that provide mechanical strength (Cosgrove & Jarvis, 2012). It is therefore apparent that cell wall components and their molecular interactions are critical in conferring the various properties that cell walls possess, but the detailed knowledge of the spatial organization of each cell wall component is missing. Gaining such detailed knowledge would lead to a better understanding of the underlying principles of cell wall architecture, allowing the prediction of cell wall properties and function.

The primary and secondary cell walls of grasses are distinct from eudicots in several ways. Most importantly, xylan is the dominant hemicellulose in grass primary walls, with mixed‐linkage glucan being the second major hemicellulose. Depending on the species, growth stage and tissue, xylan may carry a number of different substitutions. The molecule of xylan consists of a linear backbone of β‐1,4‐xylose (Xyl) residues substituted with α‐1,2‐linked (4‐*O*‐methyl)‐glucuronic acid (GlcA), α‐1,3‐linked arabinofuranose (Ara*f*), and acetylation at the *O*‐2 and/or *O*‐3 positions (Ebringerová & Heinze, 2000). Grass xylan substitution consists mainly of Ara*f*, with comparatively fewer GlcA substitutions (Ebringerová & Heinze, 2000; Tryfona et al., 2019). However, grass xylans have some unique structural features, such as Xyl*p*–Ara*f* substitutions (Ebringerová & Heinze, 2000; Tryfona et al., 2019; Wende & Fry, 1997; Zhong et al., 2022) or hydroxycinnamic acid groups, for example, feruloyl (Fer) or coumaryl (Co) groups, esterified to the *O*‐5 position of Ara*f* residues (Feijao et al., 2021; Hatfield et al., 2017; Mueller‐Harvey et al., 1986). These hydroxycinnamic groups can be esterified or etherified to lignin (Hatfield et al., 2017). The ferulation of grass xylan is known to facilitate covalent cross links of xylan chains or xylan–lignin via diferulate bridge formation (Feijao et al., 2021; Hatfield et al., 1999, 2017; Terrett & Dupree, 2019).

The arrangement of substitutions on the backbone of xylan influences how this molecule interacts with other cell wall components. In eudicots, GlcA and acetyl group substitutions are positioned on alternate Xyl residues of the xylan backbone at the *O*‐2/*O*‐3 positions (Bromley et al., 2013; Busse‐Wicher et al., 2014). It is this even pattern of decoration that allows xylan to adopt a twofold helical screw conformation. Such conformation results in all decorations being oriented on one side of the xylan chain, which could then permit xylan molecules to hydrogen bond with the hydrophilic surfaces of cellulose. Randomly modified xylan, on the other hand, adopts a threefold conformation and therefore is more flexible and does not interact with cellulose hydrophilic surfaces (Grantham et al., 2017). The even spacing of xylan decoration is a highly conserved feature and is also found in gymnosperm xylans (Busse‐Wicher, Li, et al., 2016; Martínez‐Abad et al., 2017; Pereira et al., 2017; Terrett et al., 2019). Therefore, it becomes apparent that in eudicot angiosperms and gymnosperms the pattern of xylan substitution influences the conformation of this polysaccharide and enables interaction with cellulose fibrils.

The Ara*f* substitutions and hydroxycinnamic acid modifications of grass xylan are likely to have an effect on the conformation of xylan and its interaction with both cellulose and lignin. Our recent solid‐state nuclear magnetic resonance (ssNMR) study on never‐dried *Brachypodium distachyon* tissues confirms that some xylan in grasses binds to cellulose in the twofold conformation (Duan et al., 2021). Furthermore, lignin–polysaccharide interactions in secondary cell walls of *Zea mays* (maize) revealed by ssNMR were also found to be xylan‐conformation dependent (Kang et al., 2019). It is clear, therefore, that different xylan conformations are present in grass cell walls. However, the arrangement of substitutions on grass xylan remains unknown, and so it is not yet possible to understand fully how these arrangements might influence cell wall assembly.

To investigate the arrangements of xylan decorations in commelinid monocots, we analysed the pattern of xylan substitutions on grass xylan. We demonstrate that grass cell wall contains distinct types of xylan. These xylans have different substitution patterns, can be partially separated from each other, and are widely present on cell walls. Based on these observations, we postulate that these xylans may adapt different conformations and have different interaction properties with other cell wall components.

## RESULTS

### Some Ara*f* substitutions on grass xylan are evenly spaced and GlcA decorations are clustered together

**Figure 1:**
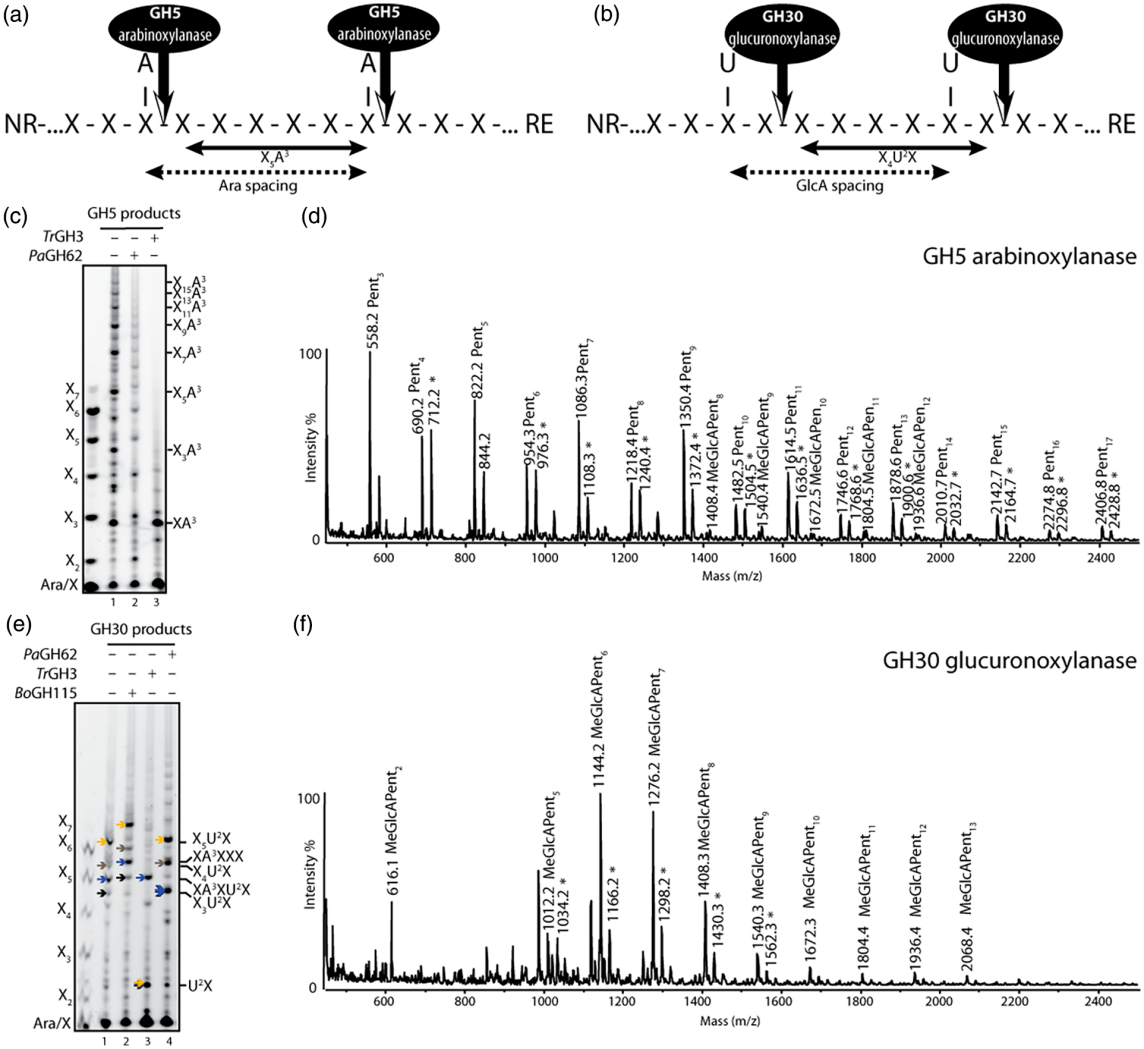
Analysis of the distribution of arabinose (Ara*f*) and glucuronic acid (GlcA) on xylan from *Miscanthus* culms. (a) Schematic representation of the GH5 arabinoxylanase action to determine Ara*f* spacing on the xylan backbone. GH5 requires Ara*f* substitutions to be α‐1,3‐linked to the active site xylose (Xyl) residue and liberates Ara*f*‐modified oligosaccharides. A, Ara*f*; NR, non‐reducing end; Pent, pentose; RE, reducing end; U, GlcA; X, Xyl. (b) Schematic representation of the GH30 glucuronoxylanase to determine the spacing of GlcA. The GH30 enzyme requires GlcA substitutions to be attached at the −2 Xyl residue from the active site and liberates singly GlcA‐substituted oligosaccharides. (c) Saponified alcohol insoluble residue (AIR) from *Miscanthus* culms was hydrolysed with GH5 arabinoxylanase and analysed by polysaccharide analysis with carbohydrate gel electrophoresis (PACE). GH5‐hydrolysed samples were further hydrolysed either with *Pa*GH62 arabinosidase or *Tr*GH3 xylosidase. Xylan oligosaccharides: X–X_7_. (d) Matrix‐assisted laser desorption/ionization time‐of‐flight mass spectrometry (MALDI‐ToF MS) spectra of 2‐AA labelled GH5 hydrolysis products. (e) Saponified AIR from *Miscanthus* culms was hydrolysed with GH30 glucuronoxylanase and analysed by PACE. GH30‐hydrolysed samples were further hydrolysed with *Bo*GH115 glucuronidase, *Pa*GH62 arabinosidase or *Tr*GH3 xylosidase. Arrows with the same colour show the flow direction of bands after sequential digestion. (f) MALDI‐ToF MS spectra of 2‐AA labelled GH30 hydrolysis products.

On *Miscanthus* culm xylan, the average frequency of Ara*f* substitutions is about 11%, whereas GlcA substitution is significantly lower, at about 5% of Xyl residues (Tryfona et al., 2019); however, it is not yet known whether Ara*f* or GlcA decoration is random or is regularly or irregularly spaced. To investigate the spacing of these substitutions, we hydrolysed xylan from *Miscanthus* culms with xylanases from two different glycosyl hydrolase (GH) families, GH5 (*Ct*GH5_34) and GH30 (*Ec*GH30) (Carbohydrate Active EnZymes database, CAZy, http://www.cazy.org; Urbániková et al., 2011). The GH5 enzyme is an arabinoxylan‐specific xylanase that displays an absolute requirement for xylans that contain Ara*f* side chains (Correia et al., 2011). This enzyme requires the Ara*f* appended to *O*‐3 of the Xyl bound in the −1 subsite (Labourel et al., 2016). By sizing the oligosaccharides released by the GH5 enzyme, the spacing between Ara*f* substitutions can be determined (Figure 1a). On the other hand, the GH30 enzyme requires GlcA substitutions to be attached at the −2 Xyl residue from the active site (Vršanská et al., 2007), and the size of the oligosaccharides released by this enzyme has been previously used to determine the spacing between two GlcA residues on the xylan backbone in Arabidopsis and gymnosperms (Figure 1b; Bromley et al., 2013; Busse‐Wicher, Li, et al., 2016).

Alcohol‐insoluble residue (AIR) from *Miscanthus* culms was depectinated, saponified and subsequently hydrolysed with the GH5 arabinoxylanase. The oligosaccharide hydrolysis products were analysed using matrix‐assisted laser desorption/ionisation (MALDI) time‐of‐flight (ToF) mass spectrometry (MS) and polysaccharide analysis by carbohydrate gel electrophoresis (PACE). A range of xylo‐oligosaccharide products of a degree of polymerisation (DP) were released, as seen by PACE (Figure 1c, lane 1). The migration differences suggested that the main series of oligosaccharides differ in DP by two pentoses, with the smallest oligosaccharide migrating slightly faster than xylotriose (X_3_). Based on the specificity of the GH5 arabinoxylanase, all xylo‐oligosaccharides should be substituted with an Ara*f* at the reducing end. Consistent with this, all the oligosaccharides were sensitive to *Pa*GH62 arabinofuranosidase (Figure 1c, lane 2). To investigate whether further substitutions were present, we treated the GH5 products with *Tr*GH3 xylosidase, which removes terminal xylosyl residues from the non‐reducing end, but is inhibited by substitutions at *O*‐3 of Xyl on the +2 subsite (Tenkanen et al., 1996). All the major GH5 oligosaccharide products were sensitive to the xylosidase, yielding one major oligosaccharide migrating slightly faster than X_3_ (Figure 1c, lane 3). The structure of this oligosaccharide was determined by NMR spectroscopy and was identified as β‐Xyl*p*‐(1→4)‐[α‐Ara*f*‐(1→3)]‐Xyl*p* (XA^3^; Figure S1; Table S1). Therefore, taking together the PACE and NMR data, the majority of the GH5 hydrolysis products have just a single Ara*f* substitution at the reducing end. This is consistent with the MALDI‐ToF‐MS spectra, which revealed mainly neutral oligosaccharides (Figure 1d). Furthermore, the dominance of oligosaccharides consisting of odd‐number pentose residues is consistent with the backbone length of even numbers of xylosyl residues, plus a single Ara*f* on the reducing end xylose. Only minor acidic oligosaccharide products were detected, modified by a single methylated GlcA (MeGlcA) residue. The results indicate that the xylan digested by the GH5 arabinoxylanase has sparse and predominantly even‐spaced Ara*f*, and very few MeGlcA residues.

Glucuronoxylanase GH30 hydrolysis of *Miscanthus* culm AIR and analysis with PACE revealed several oligosaccharides demonstrating migration between X_4_ and X_7_, plus a few minor larger oligosaccharides; two major (yellow and blue arrows; Figure 1e, lane 1) and two minor oligosaccharides (grey and black arrows; Figure 1e, lane 1) were studied further. All these oligosaccharides were sensitive to *Bo*GH115 α‐glucuronidase, indicating the presence of GlcA decoration, as expected (Figure 1e, lane 2). Three of the GH115 α‐glucuronidase products now co‐migrated with xylo‐oligosaccharide standards: X_7_, X_6_ and X_5_. Given the specificity of the GH30 glucuronoxylanase, this indicates that the products were X_5_U^2^X (yellow arrow, XXXXXU^2^X), X_4_U^2^X (grey arrow, XXXXU^2^X) and X_3_U^2^X (black arrow, XXXU^2^X), respectively. One oligosaccharide did not migrate with any standard (Figure 1e, lane 2, blue arrow), suggesting that it was further substituted. Indeed, this GH30 product was sensitive to *Pa*GH62 arabinofuranosidase (Figure 1e, lane 4). Hydrolysis of the GH30 products with GH3 β‐xylosidase resulted in U^2^X from X_n_U^2^X oligosaccharides, as well as a resistant oligosaccharide (migrating near X_5_), indicating a substitution (Figure 1e, lane 3). The positions and linkages of the substitutions of this oligosaccharide were determined by MALDI‐ToF/ToF‐MS/MS as XA^3^XU^2^X (Figure S2). The GH30 products were also investigated by MALDI‐ToF‐MS (Figure 1f), confirming that MeGlcAPent_5_, MeGlcAPent_6_ and MeGlcAPent_7_ predominated. Together, the results indicate that GH30 cleaves the xylan into short oligosaccharides and suggests most of the GlcA substitutions are clustered between five and seven backbone residues apart.

**Figure 2:**
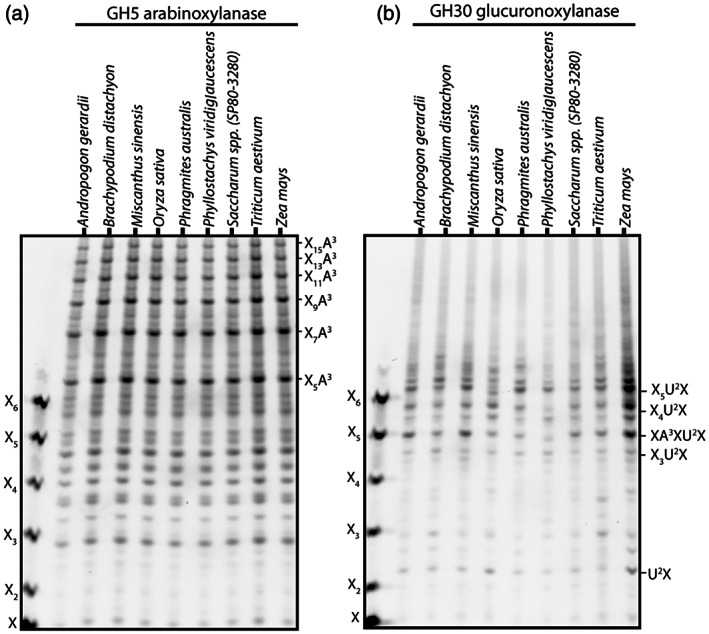
Analysis of Ara*f* and GlcA substitutions in grasses. (a) Ara*f* is regularly distributed on xylan in grasses. Saponified alcohol insoluble residue (AIR) of *Andropogon gerardii*, *Brachypodium*, *Miscanthus*, common rice, *Phragmites australis*, *Phyllostachys viridiglaucescens*, sugar cane, common wheat and maize was hydrolysed with GH5 arabinoxylanase and analysed by PACE. The major grass products correspond to substituted xylo‐oligosaccharides with Ara*f* side chains on evenly spaced Xyl residues, largely at 6‐, 8‐, 10‐, 12‐ or 14‐residue intervals. (b) GlcA substitutions are clustered together on xylan in grasses. Saponified AIR of *Andropogon gerardii*, *Brachypodium*, *Miscanthus*, common rice, *Phragmites australis*, *Phyllostachys viridiglaucescens*, sugar cane, common wheat and maize was hydrolysed with GH30 glucuronoxylanase and analysed by PACE. The major grass products correspond to substituted xylopentose XA^3^XU^2^X and xyloheptaose X_5_U^2^X.

These data revealed two quite different grass xylan substitution patterns: (i) arabinoxylan with evenly distributed Ara*f* substitutions (AXe); and (ii) glucuronoarabinoxylan with clustered GlcA modifications (GAXc). The pattern of *Miscanthus* culm xylan substitution with GlcA and Ara shown here is distinct from that found in gymnosperms and many angiosperms (Busse‐Wicher, Li, et al., 2016). To investigate whether grass substitution patterns are conserved among grass species, we produced AIR from culms of eight different grass species: *Andropogon gerardii*, *B. distachyon*, *Oryza sativa*, *Phragmites australis*, *Phyllostachys viridiglaucescens*, *Saccharum* spp. (SP80‐3280), *Triticum aestivum* and *Z. mays*. Digestion of AIR with GH5 arabinoxylanase and analysis by PACE showed that a number of oligosaccharides were released from all grass species, which co‐migrate with the typical AXe oligosaccharides released from *Miscanthus* culms (Figure 2a). Similarly, digestion of culm AIR with GH30 glucuronoxylanase and analysis by PACE showed that the oligosaccharides released also co‐migrate with the GAXc oligosaccharides released from *Miscanthus* culms (Figure 2b). The relative quantities of oligosaccharides released by either of the two enzymes varied among the different grass species analysed, which might reflect differences in development or genuine quantitative differences in the substitution of xylan (Tryfona et al., 2019). Nevertheless, the even distribution of Ara*f* substitutions and the clustering of GlcA residues on xylan are common features across all grasses analysed.

### Extraction and separation of distinct domains of grass xylan

**Figure 3:**
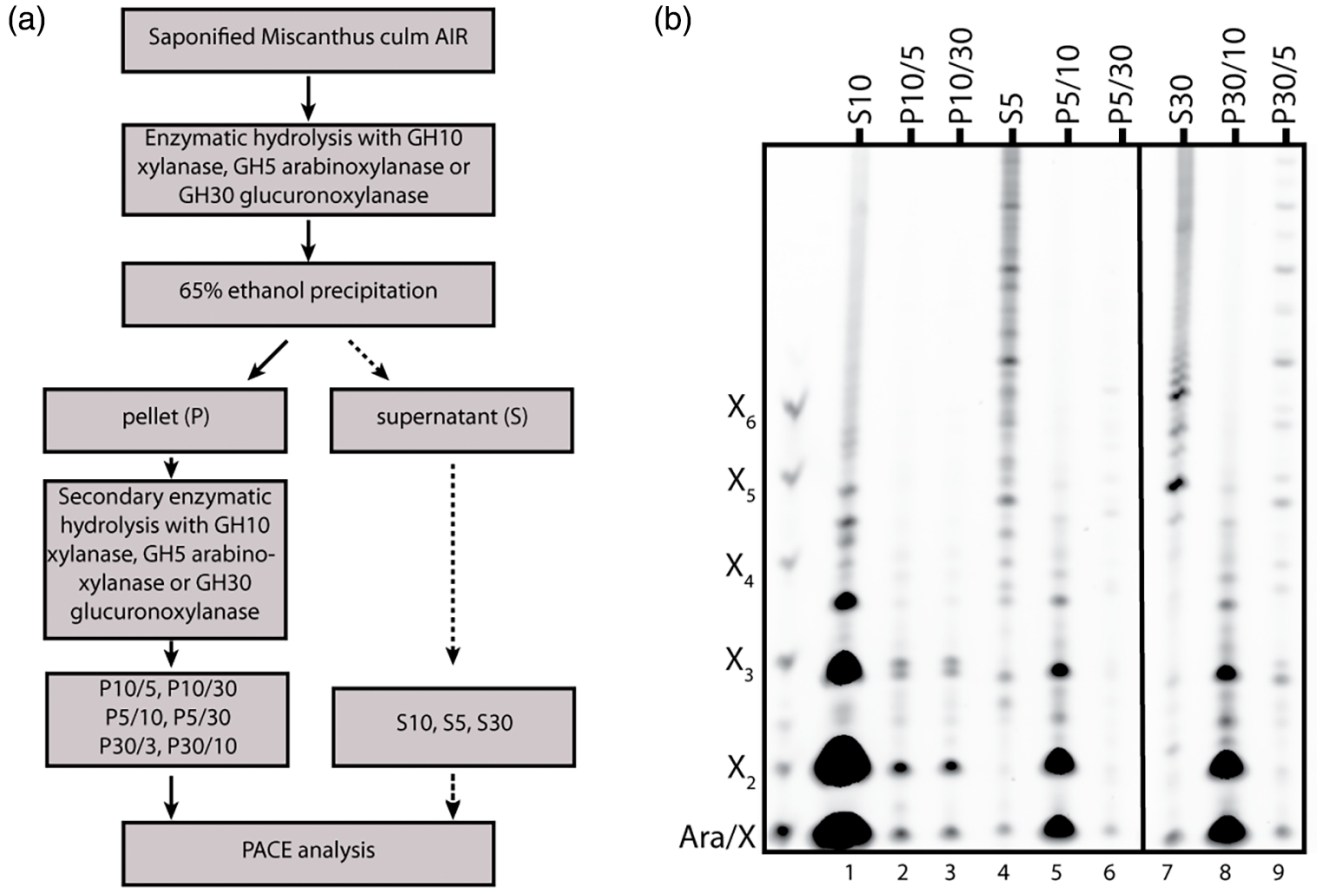
Analysis of xylanase‐accessible xylan. (a) Overview of the technical procedure used to identify xylan accessible to different xylanases. (b) Alcohol‐insoluble residue (AIR) from *Miscanthus* culms was hydrolysed with GH10, GH5 or GH30 xylanases and the undigested material was precipitated with 65% ethanol. Soluble oligosaccharides were reductively aminated with 8‐aminonaphthalene‐1,3,6‐trisulfonic acid (ANTS) and analysed by PACE (S samples). The ethanol‐precipitable pellet was incubated again with one of the remaining xylanases and soluble oligosaccharide products were analysed by PACE (P samples).

We investigated whether AXe and GAXc are distinct domains within a single type of xylan or, alternatively, represent distinct types of xylan chains. In addition, it remained unclear what proportion of xylan was constituted by these xylan types. We digested saponified *Miscanthus* culm AIR with substitution‐tolerant GH10 xylanase (Figure S3), GH5 arabinoxylanase or GH30 glucuronoxylanase. We then used 65% ethanol to separate, by precipitation, any xylanase‐resistant xylan from the released oligosaccharides. The xylan in the pellet was then treated further with each of the other xylanases (Figure 3a). This revealed significant differences in the digestibility of xylan by the three enzymes: GH10 endo‐xylanase digested all the AXe and GAXc, as the characteristic oligosaccharides were not released by these enzymes (Figure 3b, lanes 1–3). Consistent with the low GlcA substitution frequency of the AXe xylan domain, the GH30 glucuronoxylanase did not digest all the AXe (Figure 3b, lanes 7 and 9). On the other hand, the GH5 arabinoxylanase digested almost all the GAXc (Figure 3b, lanes 4 and 6), consistent with the presence of Ara substitutions in GAXc. Interestingly, a further fraction of xylan was GH5 arabinoxylanase resistant but GH10 digestible (Figure 3b, lanes 4 and 5). Thus, most patterned Ara*f* and clustered GlcA modifications are indeed found on distinct domains of xylan, AXe and GAXc, respectively. The proportions of xylan that were GH30 glucuronoxylanase resistant but GH10 digestible (Figure 3b, lane 8) and GH5 arabinoxylanase resistant but GH10 digestible (Figure 3b, lane 5) were significant, but did not represent all of the total xylan released by GH10 endoxylanase (Figure 3b, lane 1). Thus, AXe and GAXc are both relatively substantial fractions of wall xylan. Additionally, in culm cell walls there is also a small population of xylan that is digestible only by GH10 xylanase, which we call highly substituted GAX (hsGAX).

**Figure 4:**
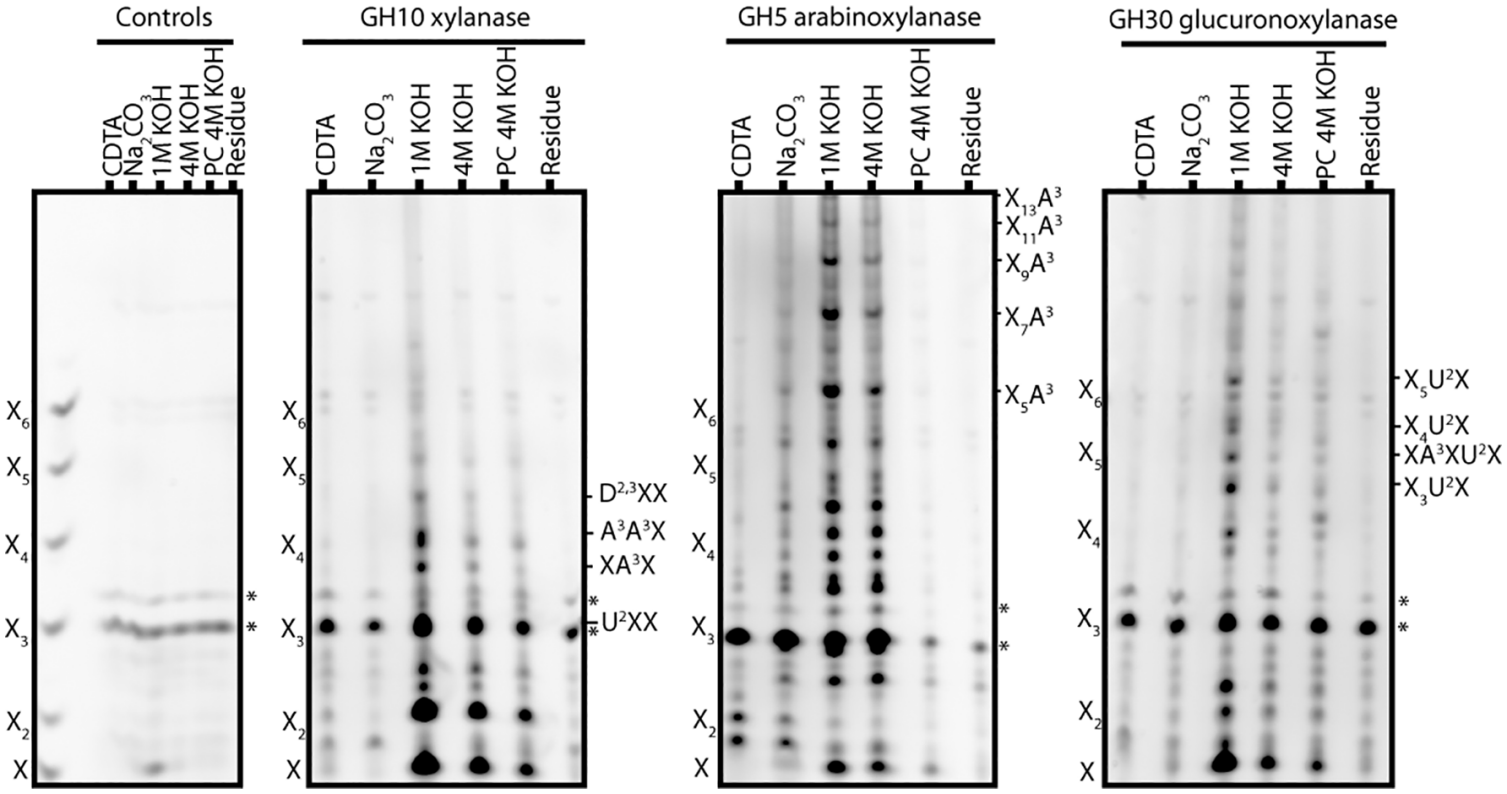
Sequential cell wall extraction from *Miscanthus sinensis* culms. Cell walls were sequentially extracted using CDTA, Na_2_CO_3_, 1 m KOH, 4 m KOH and 4 m KOH after sodium chlorite treatment (4 m KOH PC). Resulting fractions were analysed by PACE using GH10, GH11, GH30 and GH5 xylanases. Fractions not treated with enzymes are also shown. Bands marked with asterisks (*) are non‐specific labelling products.

Sequential cell wall extractions may provide information on cell wall architecture by revealing how strongly glycans are bound to cell walls. To investigate the interaction of grass xylan with other cell wall components, polysaccharides were sequentially chemically extracted from *Miscanthus* culm AIR. The solubilized polysaccharides were digested with xylanases (GH10, GH5 and GH30) and analysed by PACE (Figure 4). As expected, only trace quantities of xylan were released by 1,2‐cyclohexanediamine tetra acetic acid (CDTA) and Na_2_CO_3_ fractions, which solubilize mainly pectic polysaccharides. The majority of all types of xylan was solubilized by 1 m KOH, in accordance with previous studies (Kulkarni et al., 2012). A smaller but substantial quantity of AXe (GH5‐accessible) xylan was found in the 4 m KOH fraction. Only trace quantities of GAXc (digested by GH30 glucuronoxylanase) were released by 4 m KOH. Treatment with 4 m KOH after sodium chlorite (PC 4 m KOH) is thought to solubilize polysaccharides that are either directly or indirectly associated with lignin in the cell wall matrix (da Costa et al., 2017). A small quantity of xylan was released with 4 m KOH PC, as revealed by GH10 hydrolysis, but this was not AXe, and was probably mainly hsGAX. We conclude that harsher conditions are required for the extraction of all of the AXe compared with GAXc, and therefore AXe may be more strongly bound in the cell wall.

**Figure 5:**
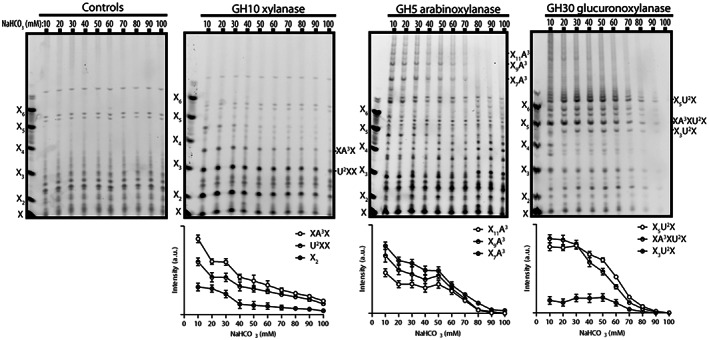
Ion‐exchange chromatography of xylan. Alcohol‐insoluble residue (AIR) from *Miscanthus* culms was depectinated with (NH₄)₂C₂O₄, and the xylan extracted by 4 m NaOH was bound to diethylaminoethanol (DEAE) cellulose and subjected to stepwise elution with increasing concentrations of NaHCO_3_ buffer (10–100 mm). The xylan in each fraction was digested with GH10, GH5 or GH30 enzymes to reveal xylan, Ara*f*‐substituted xylan or GlcA‐substituted xylan, respectively. For each hydrolysis, three oligosaccharides were selected and the band intensity was quantified by imagej to monitor the abundance of the corresponding oligosaccharides across the NaHCO_3_ buffer concentration gradient. Values are means ± standard deviations (SDs) of three independent DEAE experiments.

If GAXc and AXe are separate xylan molecule types, then they would be differently charged and separable by anion‐exchange chromatography on diethylaminoethanol (DEAE) cellulose. To investigate this question, xylan was bound to DEAE cellulose and then eluted with increasing concentrations of NaHCO_3_ (Figure 5). The presence of different types of xylan was determined by hydrolysis with GH10 xylanase, GH5 arabinoxylanase or GH30 glucuronoxylanase and analysed by PACE. GH10 xylanase hydrolysis showed xylan eluted in all fractions. Notably, AXe and GAXc eluted with somewhat differing profiles: the peak of the AXe elution was observed in the 10 mm NaHCO_3_ fraction, with lower proportions eluted in the following fractions and with only traces of the AXe diagnostic oligosaccharides detected in the 70–100 mm NaHCO_3_ fractions (the smaller oligosaccharides from xylan eluting later arise from other xylan types, perhaps including GAXc). On the contrary, substantial proportions of GAXc molecules eluted above 10 mm NaHCO_3_ fractions, suggesting a more charged molecule compared with AXe. From this we conclude that AXe and GAXc domains are found in differing proportions or perhaps are found in distinct xylan molecules.

**Figure 6:**
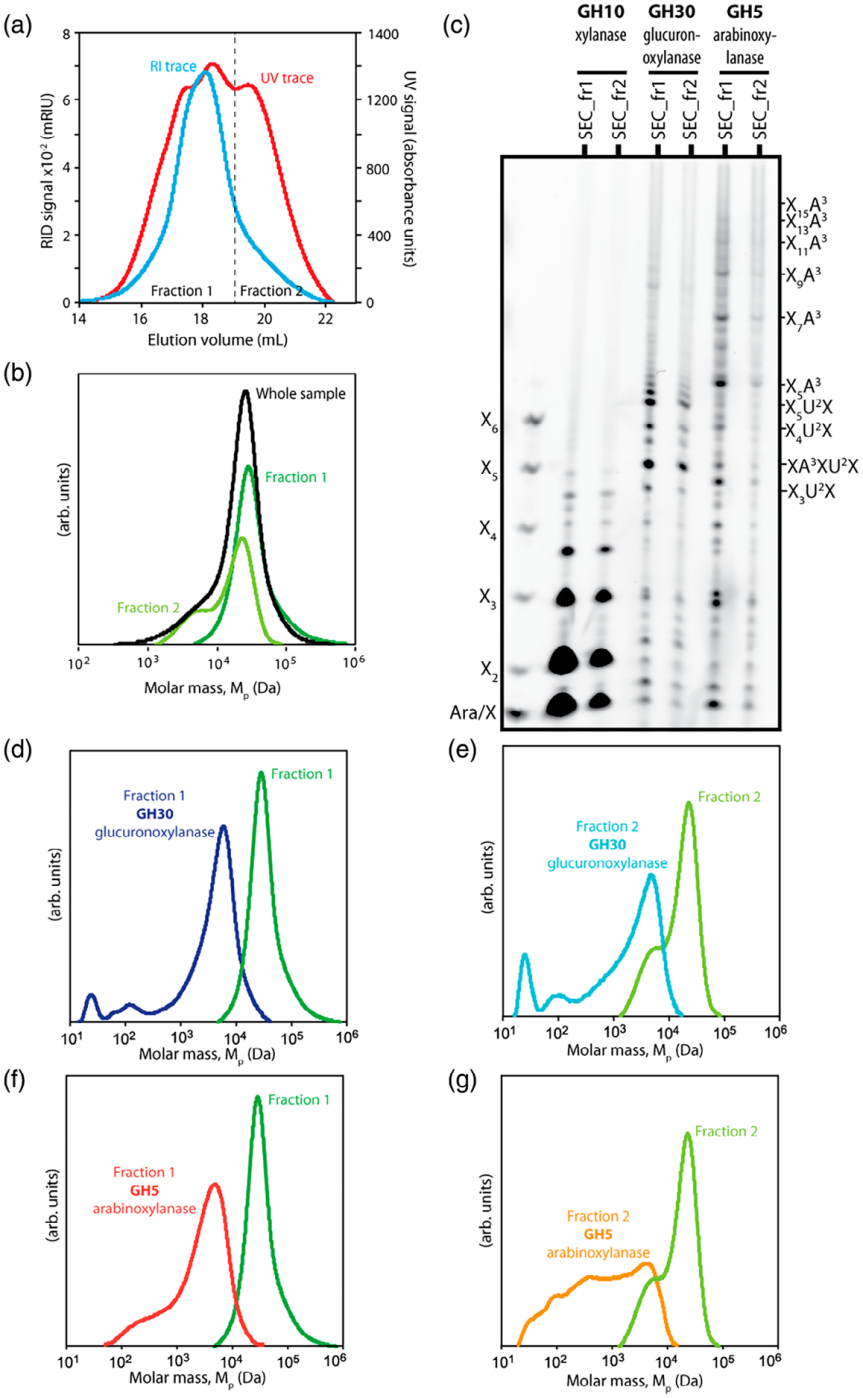
Size‐exclusion chromatography (SEC) analysis and fractionation of *Miscanthus* culm xylan. (a) Xylan extracted from *Miscanthus* culms by 4 m NaOH was analysed and fractionated by SEC using a dimethyl sulfoxide (DMSO)/LiBr elution system. The xylan eluted into two fractions: fraction 1 (collected after elution between 14 and 19 min) and fraction 2 (collected after elution between 19 and 22 min). The dashed line indicates the switch from the collection of fraction 1 to the collection of fraction 2. The elugrams represent the signals from the refractive index (RI) detector (in blue) and the UV detector (in red). (b) Molar mass distributions (w log M) of the parent (intact) xylan sample and fractions 1 and 2. Fraction 1 shows a monomodal distribution with larger macromolecular populations between 10 and 100 kDa. Fraction 2 contains significantly less populations with molar masses between 1–10 kDa and larger macromolecular populations (from fraction 1) that co‐eluted during SEC fractionation due to band broadening. (c) The xylan in each fraction was digested with GH10 xylanase, GH5 arabinoxylanase or GH30 glucuronoxylanase to show the xylan substitution pattern. The GH10 hydrolysis of fraction 1 (SEC_fr1) and fraction 2 (SEC_fr2) indicates the presence of a significant quantity of xylan in both fractions. A large proportion of GH30‐digestible xylan is present in SEC_fr1, whereas a significant quantity is also present in SEC_fr2. Similarly, most of GH5‐digestible xylan eluted in SEC_fr1, with only minor quantities eluting in SEC_fr2. (d) Xylan in fraction 1 purified by SEC was hydrolysed by GH30 glucuronoxylanase and subjected again to SEC analysis. (e) Xylan in fraction 2 purified by SEC was hydrolysed by GH30 glucuronoxylanase and subjected again to SEC analysis. (f) Xylan in fraction 1 purified by SEC was hydrolysed by GH5 xylanase and subjected again to SEC analysis. (g) Xylan in fraction 2 purified by SEC was hydrolysed by GH5 xylanase and subjected again to SEC analysis.

Next, we investigated whether it was possible to resolve the two xylan molecules based on size. Xylan was subjected to size‐exclusion chromatography (SEC) using simultaneous detection with UV (280 nm) and refractive index (RI), which detects dissolved substances non‐specifically. We performed SEC using a dimethyl sulfoxide (DMSO)/LiBr elution system to avoid aggregation phenomena during separation, which could bias the molecular mass separations (Vilaplana & Gilbert, 2010). For these experiments, *Miscanthus* culm AIR was depectinated with ammonium oxalate and xylan was extracted with 4 m NaOH. The extracted xylan was then desalted, dissolved in DMSO and applied to an SEC column. The RI profile gave a broad asymmetric peak (elution between 14 and 22 min), whereas the UV signal indicated the presence of more than one population, and hence we collected the xylan in two fractions (Figure 6a): (i) fraction 1 (elution between 14 and 19 min), which contained molecules with high molecular mass; and (ii) fraction 2 (elution between 19 and 22 min), of lower abundance, which contained smaller molecular mass polymers. The larger UV signal of fraction 2 compared with fraction 1 might arise from the presence of phenolic (lignin) components associated with this xylan population that may be resistant to the alkaline treatment with 4 m NaOH. To study the molecular mass of the SEC fractions, fractions 1 (SEC_fr1) and 2 (SEC_fr2) were individually subjected to SEC‐MALS (Figure 6b). We found that SEC_fr1 contained a single mass population centred around 30 kDa, whereas SEC_fr2 contained two populations: a population with smaller molar mass (between 1 and 10 kDa) and a significant proportion of the same material as in SEC_fr1. PACE analysis with GH10 hydrolysis confirmed that xylan was present in both SEC fractions, and that it was more abundant in SEC_fr1 (Figure 6c). SEC_fr1 contained most of the GAXc, but a significant quantity also eluted in SEC_fr2. AXe also eluted mainly in SEC_fr1, but only trace quantities were observed in SEC_fr2 (Figure 6c). Therefore, GAXc and AXe xylan molecules were not clearly separated by size, and both elute mainly in the high molecular mass fraction. GH30 (Figure 6d,e) and GH5 (Figure 6f,g) digestion resulted in cleavage of all the high molecular mass xylan in both fractions but did not reduce all the xylan to small oligosaccharides, as a major peak between 1 and 10 kDa was observed after both enzymatic treatments. The sensitivity of all the higher mass xylan to both enzymes suggests these molecules contain both GAXc and AXe. The lower molecular mass xylan was partially resistant to both enzymes. This is consistent with the presence of hsGAX that eluted mainly in the small molecular mass fraction. Monosaccharide analysis conducted with high‐performance anion‐exchange chromatography and pulsed amperometric detection (HPAEC‐PAD) is consistent with SEC_fr2 containing a proportion of more highly substituted xylan (Figure S4).

### Immunolocalization of AXe and GAXc in *B. distachyon* culms

**Figure 7:**
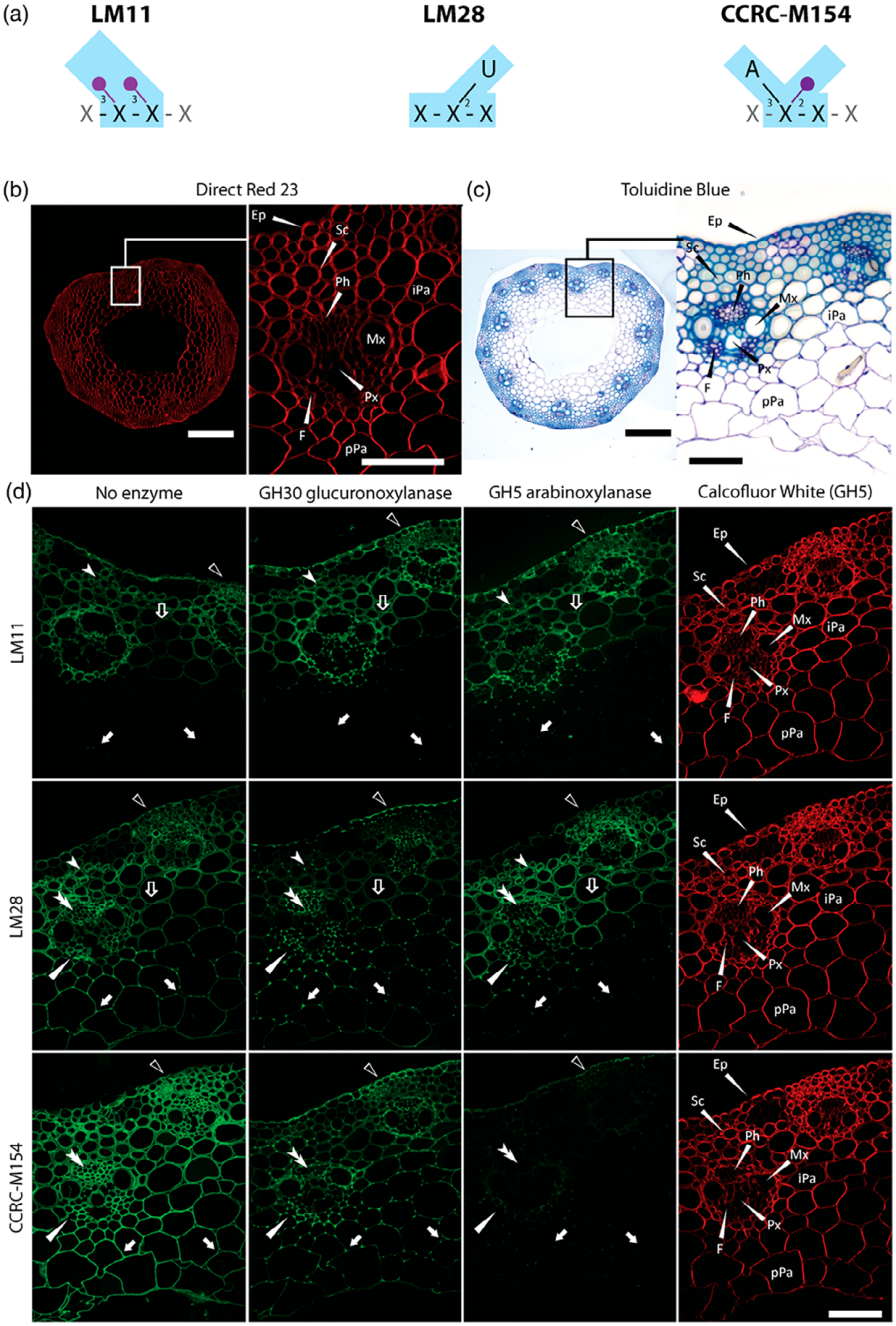
Indirect immunofluorescence detection of xylan epitopes in transverse sections of *Brachypodium distachyon* internodes before and after treatment of sections with GH30 glucuronoxylanase or GH5 arabinoxylanase. Equivalent sections were also stained with Direct Red 23 and toluidine blue to show the tissue anatomy and deposition of lignin in the walls of all cell types. (a) Schematic representation of epitopes (highlighted in light blue) of cell wall‐directed antibodies LM11, LM28 and CCRC‐M154 according to Ruprecht et al. (2017). Linkages that are marked with purple indicate positions that must not be substituted for antibody to bind. Light‐grey sugar residues and linkages indicate positions for substitutions that are allowed but not required for binding (Ruprecht et al., 2017). A, Ara*f*; U, GlcA; X, Xyl. (b) Direct Red 23 (red) showing tissue anatomy. Close‐up panel shows an individual vascular bundle and surrounding cells, secondary cell wall thickening is visible for epidermis, sclerenchyma and metaxylem cell walls. (c) Microscopic inspection of sections from culms from *Brachypodium distachyon* stained with toluidine blue to identify lignified cells. (d) Xylan immunolabelling of LR‐white serial sections with LM11 (relatively unsubstituted xylan), LM28 (GlcA‐substituted xylan) and CCRC‐M154 (Ara‐substituted xylan) MAbs, comparing the labelling pattern following no enzyme treatment, GH30 glucuronoxylanase hydrolysis or GH5 arabinoxylanase treatment, respectively. All sections underwent Na_2_CO_3_ and alkali treatment (4 m NaOH), which removed ester‐linked substituents from the cell walls, and pectate lyase treatment to enhance mAb binding. Calcofluor staining of the GH5‐treated sections are shown for all treatments as a comparison (right column). Key for arrows: empty triangles, epidermis; white arrowheads, sclerenchyma; double arrowheads, phloem; white triangles, fibres; empty arrows, interfascicular parenchyma; white arrows, pith parenchyma. Ep, epidermis; F, fibres; iPa, interfascicular parenchyma; Mx, metaxylem; Ph, phloem; pPa, pith parenchyma; Px, protoxylem; Sc, sclerenchyma. Scale bars: whole sections, 200 μm; close‐ups, 50 μm.

Monoclonal antibodies (MAbs) are versatile tools that in conjunction with fluorescent imaging can detect and localize cell wall glycans in cell walls with high sensitivity through the specific recognition of oligosaccharide structures. To investigate whether AXe and GAXc are localized in different cell types we employed three well‐characterized xylan‐directed MAbs (Figure 7a): LM11, which binds to the unsubstituted β‐1,4‐linked xylan backbone (Ruprecht et al., 2017); LM28 (Cornuault et al., 2015), which binds to a glucuronosyl‐containing epitope widely present in heteroxylans, such as GAXc; and CCRC‐M154, which selectively binds to xylan oligosaccharides with a 3‐linked Ara*f* substitution (Ruprecht et al., 2017) that should recognize AXe. We performed antibody labelling of the middle of the second internode from the base of 5‐week‐old culms of *B. distachyon* (*Brachypodium*), which were treated with 4 m NaOH and pectate lyase to improve the binding of the antibodies (Verhertbruggen et al., 2017). Transverse *Brachypodium* culm sections were either directly labelled with LM11, LM28 or CCRC‐M154 antibodies or were treated with GH30 glucuronoxylanase or GH5 arabinoxylanase prior to MAb labelling. Sections were also counterstained with Calcofluor white, which binds to cellulose and other β‐glycans and fluoresces under UV excitation, allowing a very effective visualization of cell walls (Wood, 1980). To identify lignified tissues, equivalent transverse sections (not treated with 4 m NaOH) were stained with Toluidine blue O, which labels poly‐aromatic substances such as lignin (Pradhan Mitra & Loqué, 2014).

First, we used Direct Red 23 (Anderson et al., 2009) to label cellulose and assess the vascular tissue anatomy. The labelling revealed vascular bundles of phloem cells, flanked by two large metaxylem vessels and a central protoxylem cell surrounded by sheaths of fibre cells (Figure 7b). Secondary cell wall thickening was apparent in epidermis, sclerenchyma, interfascicular parenchyma and metaxylem cells. Toluidine blue O stained these walls bright blue, indicating lignification. Phloem, sheath fibres and pith parenchyma cell walls were stained dark blue/purple and therefore were not lignified (Figure 7c). The different xylan epitopes recognized by LM11, LM28 and CCRC‐M154 were widely detected, indicating all cell walls contained xylan molecules, and at least a proportion of these have glucuronic acid and Ara*f* substitutions. However, the LM11 epitope (unsubstituted xylan) was absent from pith parenchyma and phloem cell walls and an overall reduced binding was notable for interfascicular parenchyma, epidermis and sclerenchyma regions (Figures 7d and S4). This suggests that these cell walls have relatively higher proportions of frequently substituted xylan.

We were interested to understand to what extent the LM28 anti‐glucuronoxylan labelling reflects GAXc presence. We digested the GAXc by GH30 glucuronoxylanase treatment of the sections prior to antibody labelling. Remarkably, LM28 binding was completely abolished in all cell walls, with only weak binding detected in the corner cell regions (Figure 7d). The GH30 glucuronoxylanase treatment exposed additional unsubstituted xylan epitopes for LM11 binding in all cell walls, excepting primary cell walls of pith parenchyma, unlignified cell walls of phloem, sheath fibres and protoxylem. CCRC‐M154 binding was notably reduced in all cell walls but again some labelling remained in the corner regions of the cell wall (Figures 7d and S5). These results suggest that highly substituted enzyme‐resistant xylans are distributed in the corners of cells.

The distribution of AXe was studied by GH5 arabinoxylanase treatment of sections prior to antibody labelling with CCRC‐M154. This enzyme removed labelling from all cell walls, indicating that this enzyme was very effective in digesting Ara‐substituted xylan in the sections. Similar to GH30 glucuronoxylanase treatment, GH5 arabinoxylanase treatment exposed unsubstituted xylan epitopes for LM11 binding in the epidermis, sclerenchyma, vascular bundles and interfascicular parenchyma. LM28 binding was also affected by the arabinoxylanase GH5 treatment, with complete loss of binding from epidermis, pith parenchyma, phloem, xylem and the surrounding sheath fibre cells, whereas LM28 binding was significantly reduced from sclerenchyma and interfascicular parenchyma cells (Figures 7d and S4). These results are consistent with the GAXc of epidermis, pith parenchyma, phloem and fibre sheath cells possessing the XA^3^XU^2^X sequence that is sensitive to both GH30 and GH5. The CCRC‐M154 labelling was more profoundly affected by the arabinoxylanase than the glucuronoxylanase, consistent with it labelling AXe in addition to the GAXc. Together, the demonstration of the sensitivity of MAb labelling to enzyme digestion suggests that neither AXe nor GAXc are restricted to certain cell types and are found in cells with primary and with secondary cell walls.

## DISCUSSION

There is increasing interest in the arrangement of substitutions along the xylan backbone and the resulting implications for the interaction of this polysaccharide with cellulose and lignin. It has been demonstrated that there is a precise patterning of some xylan substitutions, not only in eudicots (Bromley et al., 2013; Busse‐Wicher et al., 2014; Vršanská et al., 2007) but also in gymnosperms (Busse‐Wicher, Li, et al., 2016; Martínez‐Abad et al., 2017). In certain plant groups, patterning has been observed for xylan acetylation as well as for glucuronidation. This very specific spacing may facilitate the interaction of twofold screw xylan with the hydrophilic surface of the cellulose microfibrils of plant cell walls (Busse‐Wicher et al., 2014; Busse‐Wicher, Grantham, et al., 2016; Busse‐Wicher, Li, et al., 2016; Grantham et al., 2017; Gupta et al., 2021; Kang et al., 2019; Martínez‐Abad et al., 2017). Here, we show that the precise patterning is even more widely spread in vascular plants, by extending these observations to new patterns and plant groups: the arabinosylation of xylan and grasses. We further demonstrate that grass cell wall xylan consists of distinct types with different substitution patterns that are widely present in cell walls. We hypothesize that these xylan populations in grasses reflect functional specialization and interact differently with cellulose and other cell wall components.

We studied the spacing of Ara*f* and GlcA substitutions along the xylan backbone. Interestingly, we found a xylan fraction with an even number of Xyl*p* residues between each Ara*f*, with the spacing ranging from two to at least 16 Xyl*p* residues apart. As this arabinoxylan has evenly spaced Ara*f* substitutions, we named it AXe. In contrast, to the even spacing of Ara*f*, GlcA substitutions were detected predominantly spaced five, six or seven Xyl residues apart. One of the products of GH30 glucuronoxylanase was found to carry an Ara*f* substitution. We named this glucuronoarabinoxylan with clustered GlcA, GAXc. The pattern of Ara*f* and GlcA substitutions was found to be remarkably similar in all grass species analysed, supporting the view that there is a relatively precise, non‐random arrangement of the substitutions. Our finding of the regular patterning of Ara*f* and GlcA substitutions in grass xylan is consistent with earlier studies giving evidence of a repetitive structure in grass xylan (Carpita et al., 2001; Faik, 2010; Gruppen et al., 1993; Kozlova et al., 2012; Zeng et al., 2010).

### AXe and GAXc are widely present in cell walls and are not always found on separate molecules

Our finding of the structurally different domains of xylan raised the question of whether the domains of xylan are present on the same or distinct molecules and whether they are present in specific types of cell walls, such as primary and secondary cell walls. We found that both AXe and GAXc represent substantial proportions of cell wall xylan. To separate any different populations of xylan from *Miscanthus* culms, we performed sequential cell wall extraction, ion exchange and SEC. The separation profiles provided evidence for the somewhat differing extractability of xylan domains from the cell wall and differently charged xylan populations. SEC separation resulted into a low molecular weight and a more abundant high molecular weight xylan population containing the majority of both AXe and GAXc. The low‐molecular‐weight fraction might contain mostly hsGAX, as it was most easily digestible with GH10 xylanase. On the other hand, the high‐molecular‐weight fraction was sensitive to both GH5 arabinoxylanase and GH30 glucuronoxylanase, suggesting that these high‐molecular‐weight molecules contain both AXe and GAXc. Both SEC fractions absorbed UV. Therefore, we speculate that AXe and GAXc are: (i) two distinct molecules that are cross linked with alkali‐resistant phenolic–polysaccharide ether bonds (Burr & Fry, 2009); or (ii) contiguous domains contained within the same xylan molecule that is covalently linked to lignin through alkali‐resistant benzyl ether bonds (Iiyama et al., 1990; Lam et al., 2003; Martínez‐Abad et al., 2018), forming lignin–carbohydrate complexes.

Our data on the sequential chemical extraction of xylan from cell walls of *Miscanthus* culms are consistent with previous studies on cell walls of various grass species (Kulkarni et al., 2012). Xylans with varying degrees of substitution have been reported for grasses: a GAX with a high degree of Ara*f* substitution (hsGAX) and a GAX with a low degree of substitution (lsGAX) (Shrestha et al., 2019; Tabuchi et al., 2011; Wang et al., 2014, 2016). These xylans are reported to interact differently with cellulose: hsGAX is thought to be more mobile than lsGAX and is found in the interstitial matrix, playing the role of a ‘filler’, whereas the more rigid, relatively unsubstituted lsGAX is tightly associated with cellulose microfibrils (Wang et al., 2016). Consistent with these findings, Kozlova et al. (2014) described three domains of GAX in maize roots: one keeping cellulose microfibrils apart, one interacting with them and a middle domain between the two, which links them. It is therefore conceivable that some grass GAX is tightly adherent to cellulose microfibrils and other cell wall components, providing mechanical strength, whereas other GAX keeps cellulose microfibrils separated, which would give cell walls plasticity and allow them to expand.

Using fluorescence microscopy with MAb probes, and arabinoxylan and glucuronoarabinoxylan‐specific degrading enzymes, we found that AXe and GAXc molecules are widespread in walls of different cell types of *Brachypodium* culm. This supports a role for these different xylans in the same walls, across many cell types. We found that pith parenchyma, phloem and sheath fibre cell walls were distinctive, as they showed no LM11 epitopes (unsubstituted xylan) but had relatively abundant levels of LM28 (uronic acid substituted) and CCRC‐M154 (3‐Ara*f* substituted). These walls do not have secondary thickening and are not lignified, so these antibody labelling results suggest that unlignified tissues contain more highly substituted xylan. This is consistent with earlier findings on maize culms (Suzuki et al., 2000): low‐substituted GAX was found in lignified walls and highly substituted GAX was found in unlignified and primary cell walls at an early developmental stage. Although our probes are likely to have revealed the most abundant xylan molecules, it is also possible that there is masked xylan in the culm sections not initially bound by the antibodies or cleaved by the hydrolase enzymes. Some LM11 epitopes, for example, were revealed after GH5 and GH30 hydrolysis, whereas both LM28 and CCRC‐M154 epitopes localized strongly in cell wall corner areas after GH30 enzymatic treatment.

Recently, transcriptome and immunocytochemistry analysis during the elongation growth of maize roots showed that homologous members of glycosyltransferase families involved in GAX biosynthesis were expressed differently during the early and late stages of development, suggesting that two separate sets of genes may be responsible for the biosynthesis of GAX for primary and secondary cell wall deposition. Glycosyltransferases (GTs) involved in the synthesis of secondary cell wall GAX from GT61 (XAX, MUCI) and GT8 (GUX1 and GUX2) families were widely present at late stages of development (Kozlova et al., 2020).

### Structural variation may reflect functional specialisation and distinct interactions with wall components

**Figure 8:**
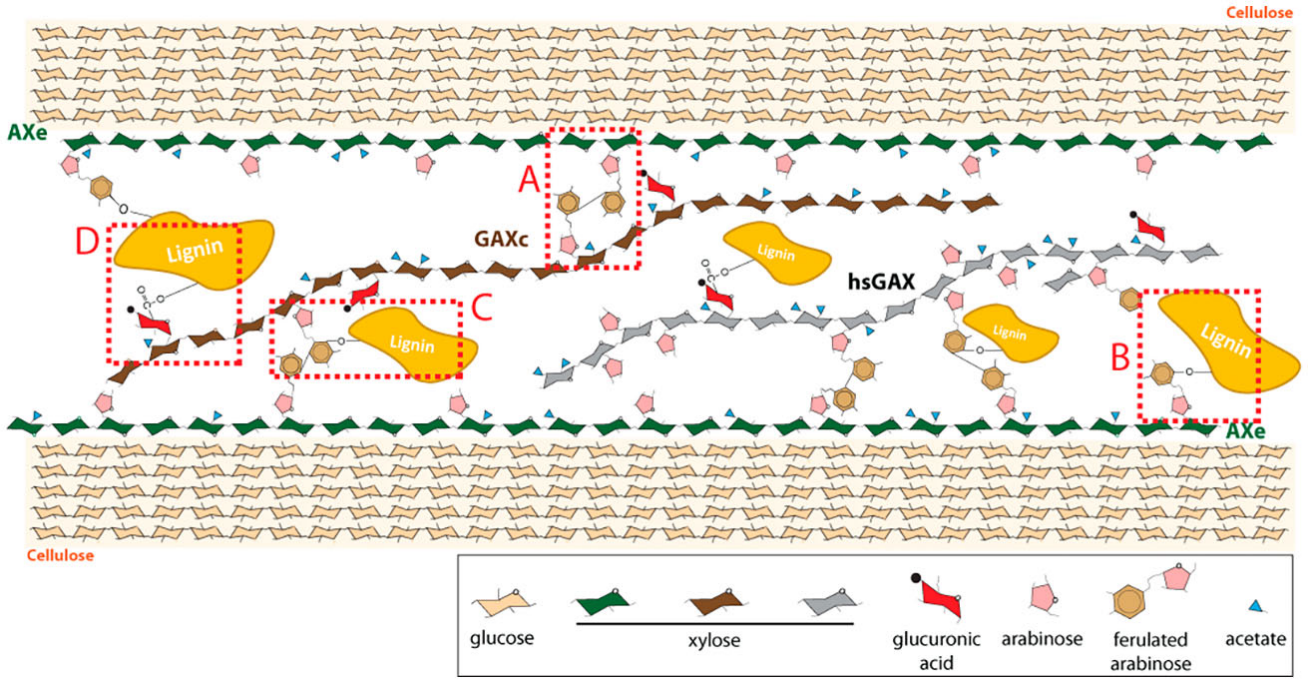
Molecular model indicating possible interactions of different grass secondary cell wall xylans with other cell wall components. AXe (green chair conformations), with an even distribution of Ara*f* residues, could adopt a twofold conformation and tightly associate with the hydrophilic surface of cellulose (orange chair conformations). Some Ara*f* residues could be feruloylated and therefore AXe could cross‐link with other xylan chains (box A) or lignin (box B). GAXc (brown chair conformations), with clustered distribution of GlcA substitutions, is hypothesized to adopt a threefold helical screw conformation, and if some of Ara*f* residues are feruloylated may tether adjacent xylan chains (box A) or cross‐link to lignin (box C). GAXc could also cross‐link to lignin via GlcA substitutions (box D). hsGAX (grey chair conformations) is most likely to be situated in the matrix and plays the role of “space filler”. For simplicity, AXe and GAXc are shown as separate molecules. Additional phenolic–polysaccharide or phenolic–lignin ether linkages are possible.

Here, we propose that the structurally different AXe, GAXc and hsGAX molecules might have distinct interactions with cellulose and lignin, reflecting functional specialization, and speculative linkages and roles are shown in Figure 8. We hypothesize that an even distribution of the hydrophilic Ara*f* residues on the AXe backbone would favour a twofold conformation of the xylan molecule. Molecular dynamics simulations predict evenly decorated xylan would adopt a twofold helical screw conformation upon interaction with the hydrophilic cellulose surface (Busse‐Wicher et al., 2014; Gupta et al., 2021). We do not yet know whether the AXe is acetylated, as our samples were alkali‐extracted, but an even pattern of acetylation could further promote the xylan–cellulose association (Gupta et al., 2021; Pereira et al., 2017). Furthermore, our recent ssNMR study on never‐dried *B. distachyon* tissues confirms that some xylan in grasses has branches that permit binding to cellulose in the twofold conformation (Duan et al., 2021). Indeed, this xylan is preferentially acetylated, suggesting that AXe might be more highly acetylated than other xylans in grasses.

Some Ara*f* residues on grass xylan are modified with ferulic acid (Feijao et al., 2021; Mueller‐Harvey et al., 1986). Strong evidence indicates that xylan in the secondary walls of grasses is cross‐linked to lignin by the extensive copolymerization of their ferulates (de Oliveira et al., 2015; Feijao et al., 2021; Grabber et al., 1995; Grabber & Lu, 2007; Ralph, 2010). Ferulic acid has also been described to cross‐link xylan chains through oxidative coupling with other ferulic acid groups (Feijao et al., 2021; Ishii, 1997; Ralph, 2010). Hence, we hypothesize that AXe could be cross‐linked either with other xylan molecules or with lignin via diferulate bridges (Figure 8, boxes A and B, respectively).

We postulate that clustered GlcA substitutions on GAXc would obstruct the interaction of this molecule with the hydrophilic cellulose surface through steric hindrance (Busse‐Wicher et al., 2014; Grantham et al., 2017). Therefore, this type of xylan would adopt a threefold helical screw conformation, would extend into the matrix and would interact with other cell wall components, such as lignin (Figure 8). Indeed, recent ssNMR data have indicated that lignin could preferentially bind xylans with threefold or distorted twofold helical screw conformations that are not closely associated with cellulose (Kang et al., 2019). As GAXc is also modified with Ara*f* residues, we hypothesize that some of these residues could be feruloylated. Therefore, GAXc could covalently cross‐link to lignin or other xylan chains through ferulic acid (Figure 8, box C). Hardwood glucuronoxylan can be esterified to lignin via its GlcA substituents (Nishimura et al., 2022). It is therefore plausible that some of the GlcA residues on GAXc are esterified to lignin (Figure 8, box D).

hsGAX is probably in a threefold screw conformation because of the dense substitutions and is likely to be found in the matrix. This hypothesis is consistent with several studies suggesting that a type of highly substituted mobile GAX (hsGAX) is found in the interfibrillar space and plays the role of a ‘filler’, whereas the relatively unsubstituted GAX is more tightly associated with cellulose microfibrils (Carpita et al., 2001; Kozlova et al., 2014; Wang et al., 2014, 2016) and contributes to cell wall strength and mechanics, functioning as a cementing substance (Tabuchi et al., 2011).

## CONCLUSION

Over the last few years there has been an increasing number of studies into the patterning of decoration of xylan and the implications this patterning has on interactions with other cell wall components. Here, we demonstrated that the even spacing of decorations is highly conserved in plants and can be found not only in Arabidopsis (Bromley et al., 2013; Busse‐Wicher et al., 2014), hardwoods (Busse‐Wicher, Li, et al., 2016; Derba‐Maceluch et al., 2015; Vršanská et al., 2007) and softwoods (Busse‐Wicher, Li, et al., 2016), but also in grasses. Furthermore, we demonstrated that distinct xylan molecules are found in walls of many cell types, suggesting the functional specialization of xylan within individual walls. Our work highlights the need of xylan biosynthesis‐related genetic resources in plants such as *B. distachyon* for the detailed study of grass xylans, as it will be important to study the function of different xylans by creating mutants in the different xylan types. The engineering of grass xylan could result in biomass with distinctive and optimized properties for use in a more sustainable bioeconomy.

## EXPERIMENTAL PROCEDURES

### Plant material

The plant materials used in this study were fresh stem material from plants aged 2.5–3.0 months: *Andropogon gerardii* (collected from the University of Cambridge Botanic garden), *B. distachyon* (Brachypodium; grown in the University of Cambridge glasshouse), *Miscanthus sinensis* (*Miscanthus*; from Luisa Trindade and Oene Dolstra, Wageningen University, the Netherlands), *O. sativa* (rice; grown in the University of Cambridge glasshouse), *Phragmites australis* (collected from the University of Cambridge Botanic garden), *Phyllostachys viridiglaucescens* (bamboo; collected from the University of Cambridge Botanic garden), *Saccharum* spp. SP80‐3280 (sugar cane; from Marcos Buckeridge, University of Sao Paulo, Brazil,), *T. aestivum* (wheat; from Greg Tucker, University of Nottingham, UK) and *Z. mays* (maize; grown in the University of Cambridge glasshouse).

### Extraction of AIR

Plant culms were harvested, submerged in 96% (v/v) ethanol and boiled at 70°C for 30 min to inactivate the enzymes. Following homogenization using a ball mixer mill (Glen Creston, now Retsch, https://www.retsch.com), the pellet was collected by centrifugation (4000 ***g*** for 15 min) and was washed with 100% (v/v) ethanol, twice with chloroform:methanol (2:1), followed by successive washes with 65% (v/v), 80% (v/v) and 100% (v/v) ethanol. The remaining pellet of AIR was air dried.

### Hemicellulose extraction

AIR preparations (100 mg) were depectinated in ammonium oxalate (0.5% w/v) at 85°C for 2 h, washed with H_2_O and subjected to alkali extraction in 4 m NaOH for 1 h at room temperature (RT) (21°C). NaOH was removed from the extracted xylan in the supernatant by passing through a PD‐10 column (GE Healthcare, https://www.gehealthcare.com) and the recovered xylan was kept in aliquots for further analysis.

### Enzymatic hydrolysis and enzymes

The enzymes used in this study were: GH10 endo‐β‐1,4‐xylanase *Cj*GH10 from *Cellvibrio japonicus* (Charnock et al., 1998); GH115 α‐glucuronidase *Bo*GH115 from *Bacteroides ovatus* (Rogowski et al., 2014; Ryabova et al., 2009); GH62 α‐arabinofuranosidase *Pa*GH62 from *Penicillium aurantiogriseum* (Peng et al., 2017) and GH3 β‐1,4 xylosidase *Tr*GH3 from *Trichoderma reesei* (Margolles‐Clark et al., 1996), both gifts from Novozymes (https://www.novozymes.com); endo‐glucuronoxylanase *Ec*GH30 from *Dickeya dadantii* (formerly known as (*Erwinia chrysanthemi*) (Urbániková et al., 2011); and endo arabinoxylanase *Ct*GH5 from *Clostridium thermocellum* (Correia et al., 2011), a gift from Professor Harry Gilbert (Newcastle University, UK). All enzymes were added at a final concentration of 2 μm and incubated at 21°C (40°C for *Ct*GH5) under constant shaking for 24 h.

Five hundred micrograms of extracted xylan was used for enzymatic hydrolysis. Xylan was hydrolysed in 50 mm ammonium acetate buffer, pH 6.0 (pH 4.0 for *Ct*GH5), overnight before boiling for 30 min to heat‐inactivate the enzymes. After digestion, samples were taken to dryness *in vacuo* before further processing, depending upon the analytical technique used.

For the analysis of xylanase accessible xylan, enzymatic hydrolyses were stopped by the addition of three reaction volumes of 96% (v/v) ethanol before precipitation of the undigested substrates and separation of the reaction products for further enzymatic hydrolysis.

### Polysaccharide analysis by carbohydrate gel electrophoresis

The derivatization of carbohydrates was performed according to previously developed protocols (Goubet et al., 2002). Carbohydrate electrophoresis and PACE gel scanning and quantification was performed as described by Goubet et al. (2002, 2009). Control experiments without substrates or enzymes were performed under the same conditions to identify any non‐specific compounds in the enzymes, polysaccharides/cell walls or labelling reagents.

### Preparation, reductive amination, purification of xylooligosaccharides for MALDI‐MS and MALDI‐MS/MS CID analysis

Xylooligosaccharides were desalted using HyperSep Hypercarb cartridges (Thermo‐Hypersil‐Keystone, part of ThermoFisher Scientific, https://www.thermofisher.com), as previously described (Tryfona et al., 2019). Oligosaccharides were lyophilized and then reductively aminated with 2‐AA (Sigma‐Aldrich, https://www.sigmaaldrich.com) before being purified from the reductive amination reagents using a Glyko Clean S cartridge (Prozyme, now Agilent, https://www.agilent.com), as previously described (Tryfona & Stephens, 2010). One microliter of reaction supernatant was mixed with an equal volume of 20 mg ml^−1^ 2,5‐dihydroxybenzoic acid (Sigma‐Aldrich) matrix in 50% methanol containing 0.4 mg ml^−1^ ammonium sulphate ((NH_4_)_2_SO; Enebro & Karlsson, 2006) and was spotted on an MTP 384 ground steel target plate (Bruker, https://www.bruker.com). The spotted samples were then air dried before being analysed on an UltrafleXtreme MALDI‐TOF/TOF mass spectrometer (Bruker). MALDI‐CID spectra were acquired with an average 50 000 laser shots per spectrum. The oligosaccharide ions were allowed to collide in the CID cell with argon at a pressure of 7.2 × 10^−6^ T.

### Solution NMR analysis of *Ct*GH5 dependent trisaccharide

Depectinated, saponified *Miscanthus* culm AIR (250 mg) was hydrolysed with *Ct*GH5 arabinoxylanase, followed by *Tr*GH3 β‐1,4‐xylosidase, using the enzyme hydrolysis conditions described above. The resulting oligosaccharide mixture was then fractionated using HyperSep Hypercarb cartridges (Thermo‐Hypersil‐Keystone) with increasing acetonitrile gradient. Consequently, fractions containing the *Ct*GH5‐dependant trisaccharide were pooled, solubilized in 0.6 ml of D_2_O and analysed by NMR. NMR spectra were recorded at 298 K with a Bruker AVANCE III spectrometer operating at 600 MHz equipped with a TCI CryoProbe. Two‐dimensional ^1^H‐^1^H TOCSY, ROESY, ^13^C HSQC, HSQC‐TOCSY and H2BC experiments were performed, using established methods (Cavanagh et al., 1996; Nyberg et al., 2005); the mixing times were 70 and 200 msec for the TOCSY and ROESY experiments, respectively. Chemical shifts were measured relative to internal acetone (δH = 2.225, δC = 31.07 ppm). Data were processed using azara 2.8 (copyright 1993–2022, Wayne Boucher and Department of Biochemistry, University of Cambridge, unpublished) and chemical‐shift assignment was performed using analysis 2.4 (Vranken et al., 2005).

### Reducing‐end reduction

The reducing end was reduced with 10 mg ml^−1^ NaBH_4_ in 0.5 m NaOH for 2 h at RT. The reaction was terminated by adjustment to pH 5.5 with glacial acetic acid on ice. The samples were desalted by passing through a PD‐10 column (GE Healthcare).

### Cell‐wall sequential fractionation

Cell‐wall sequential fractionation was based upon the methods described by Pattathil et al. (2012), with minor alterations. AIR (200 mg) was suspended in 10 ml of 0.05 m CDTA (pH 6.5) for 24 h at RT. The suspension was centrifuged (48 000 ***g***) and the pellet washed once with distilled H_2_O. The supernatants were combined as the CDTA‐soluble fraction. The AIR was subsequently extracted using increasingly harsher chemicals: 0.05 m Na_2_CO_3_ containing 0.01 m NaBH_4_ for 24 h at 4°C (Na_2_CO_3_‐soluble fraction), 1 m KOH containing 0.01 m NaBH_4_ for 24 h at RT (1 m KOH‐soluble fraction), 4 m KOH containing 0.01 m NaBH_4_ for 24 h at RT (4 m KOH‐soluble fraction) and then acidic sodium chlorite (3 mg ml^−1^ for 2 h at 80 °C) followed by 4 m KOH (4 m KOH post chlorite‐soluble fraction) containing 0.01 m NaBH_4_ for 24 h at RT. All fractions were filtered through a GF/C glass fibre filter (Whatman, now Cytiva, https://www.cytivalifesciences.com). The Na_2_CO_3_ and KOH fractions were also chilled on ice and adjusted to pH 5.0 with glacial acetic acid. All cell‐wall fractions were then dialysed extensively against deionized H_2_O for 5 days, and then lyophilized. Samples were further desalted by passing through a PD‐10 column (GE Healthcare) before further analysis.

### Ion‐exchange chromatography

Ten milligrammes of depectinated, saponified and desalted AIR (as described above) were mixed with activated DEAE DE 32 cellulose (Whatman) in 10 mm NaHCO_3_, pH 6.0. The suspension was loaded onto chromatography cartridges (Bio‐Rad, https://www.bio‐rad.com), and the flow through collected. Bound xylan was eluted buffer of increasing ionic strength of NaHCO_3_. Aliquots of each sample were hydrolysed with *Ct*GH5, *Ec*GH30 or *Cj*GH10 and analysed by PACE. Three representative oligosaccharides from each hydrolysis were selected, with signal intensity for three independent PACE analyses calculated with imagej and the mean of these three independent replicas was calculated.

### Size‐exclusion chromatography

The molar mass distributions of the xylan extracts from depectinated, saponified *Miscanthus* culm AIR (100 mg) were analysed by size‐exclusion chromatography (SECcurity 1260; Polymer Standards Services, https://www.pss‐polymer.com) coupled in series to a multiple‐angle laser light scattering detector (MALLS; BIC‐MwA7000; Brookhaven Instruments, https://www.brookhaveninstruments.com), a refractive index (RI) detector (SECcurity 1260; Polymer Standards Services) and a UV detector (SECcurity 1260; Polymer Standards Services) at a wavelength of 280 nm, as previously described (Morais de Carvalho et al., 2017). All SEC analyses were performed at a flow rate of 0.5 ml min^−1^ using DMSO (HPLC grade; Sigma‐Aldrich) with 0.5% w/w LiBr (ReagentPlus) as a mobile phase, using a column set consisting of a GRAM PreColumn, 100 and 10 000 analytical columns (Polymer Standards Services) held by thermostat at 60°C. Prior to the analyses, the xylans were dissolved directly in the SEC eluent for 16 h at 60°C. For the size fractionation of the xylan, a concentration of 10 g L^−1^ was injected repeatedly (five times), and the fractions (SEC_fr1 and SEC_fr2) were separated manually after elution and pooled together for further analysis. Each fraction was analysed individually at a concentration of 1 g L^−1^ using the same set‐up as for the fractionation. Standard calibration was performed by the injection of Pullulan Standards of known molar masses provided by the Polymer Standards Services.

### Trifluoroacetic acid hydrolysis and HPAEC‐PAD monosaccharide analysis

Samples (200 μg) were hydrolysed in 2 m trifluoroacetic acid for 1 h at 120°C. Trifluoroacetic acid was removed by evaporation under vacuum. Following resuspension in 200 μl of water, the monosaccharide sugars were separated as previously described (Tryfona et al., 2012) on a Dionex ICS3000 system equipped with a PA20 column, a PA20 guard column, and a borate trap (Dionex, now ThermoFisher Scientific). In particular, for the separation of neutral sugars, the initial isocratic wash with 12 mm KOH from 0 to 5 min followed a linear gradient from 5 to 20 min, down to 1 mm KOH. Finally, an isocratic wash step in 100 mm KOH was maintained for 5 min (from 20 to 25 min). The post‐column addition of 100 mm KOH was performed to increase PAD sensitivity.

### Histology staining and *in situ* immunolabelling

Approximately 4‐mm‐long culm sections of 5‐week‐old *B. distachyon* were dissected with razor blades and immediately fixed in a fixative solution consisting of paraformaldehyde (4% v/v; Sigma‐Aldrich) with glutaraldehyde (0.5% v/v; Sigma‐Aldrich) in phosphate‐buffered saline (PBS). Fixation, dehydration, resin infiltration and antibody washing steps were all preformed in a PELCO Biowave®Pro with SteadyTemp™ (Ted Pella, https://www.tedpella.com). Fixation was microwave radiation assisted at 150 W, under vacuum (20 Hg) for 1 min (five times) and samples were incubated in fixative solution overnight at 4°C. Accordingly, samples were washed three times with PBS buffer before culms were aligned and embedded in 1% agarose in PBS buffer. Samples were dehydrated with a graded ethanol series and infiltrated (LR White medium grade; Agar Scientific, https://www.agarscientific.com) through increasing resin concentration assisted by microwave radiation: 150 W; vacuum 20 Hg for 5 min. Resin was polymerized at 60°C during 17 h. Transverse semi‐thin sections (1 μm) were then obtained with an EM UC7 ultra‐microtome (Leica, https://www.leicabiosystems.com) and laid on SuperFrost Ultra Plus (ThermoFisher Scientific) microscopy slides.

Histology assessment through Direct Red 23 labelling was performed by mounting the sections in a solution consisting of a 1:1 solution of AF1 antifading (Citifluor, https://www.citifluor.com) with PBS containing 0.1 mg ml^−1^ of Direct Red 23 (Sigma‐Aldrich). Images were then acquired by confocal laser scanning microscopy (LSM700; Zeiss, https://www.zeiss.com).

All toluidine blue stained sections were incubated with 0.02% aqueous solution of toluidine blue O for 5 min at RT. Accordingly, samples were washed five times with water, transferred onto a microscope slide and observed with a Zeiss Axiomager.M2 under bright‐field lighting.

Prior to immunolocalization, all sections were treated with fresh sodium carbonate (Na_2_CO_3_; 0.1 m) for 2 h at RT, according to previously established protocols (Marcus et al., 2010; Xue et al., 2013). Na_2_CO_3_ was removed by washing five times with water, and sections were then incubated with 4 m NaOH for 1 h at RT. Subsequently sections were washed five times with water. The removal of pectic homogalacturonan to enhance antibody binding was carried out using pectate lyase (*Aspergillus* sp.; Megazyme) in 50 mm 3‐(cylcohexylamino)‐1‐propanesulfonic acid, 2 mm CaCl_2_ buffer, pH 10, at 25 μg ml^−1^ for 2 h at RT. Subsequently, samples were washed three times with 50 mm ammonium acetate, pH 6.0, buffer. In some cases, culm sections were pre‐treated, prior to immunolabelling, with enzymes to remove specific cell wall polysaccharides. The removal of xylan was carried out using *Ct*GH5 in 50 mm ammonium acetate buffer, pH 7.0, at 40°C for 24 h and *Ec*GH30 in 50 mm ammonium acetate buffer, pH 6.0, at 21°C for 24 h. Control sections not treated with enzymes (*Ct*GH5 or *Ec*GH30) were incubated for an equivalent time with the corresponding buffers alone. Following enzymatic treatment, sections were washed with PBS three times, then incubated for 1 h with 2% bovine serum albumin protein/PBS (BSA/PBS) to prevent non‐specific binding, and then washed for 5 min with PBS. The MAbs used in this study were the rat MAbs LM11 and LM28 and the mouse MAb CCRC‐M154. LM11 (Ruprecht et al., 2017) antibody binds to unsubstituted xylan, LM28 antibody (Cornuault et al., 2015) is directed to a [Me]GlcA‐containing epitope on heteroxylan and CCRC‐M154 (Ruprecht et al., 2017) antibody binds strongly to 3‐substituted arabinofuranoses on xylan. Primary MAbs in 2% BSA/PBS buffer were incubated overnight at 4°C, with 15‐fold dilutions for LM11 and LM28 and 1‐fold dilution for CCRC‐M154. Sections were then washed five times with 2% BSA/PBS buffer before secondary antibodies, anti‐rat IgG‐FITC (Sigma‐Aldrich) and anti‐mouse IgG‐Alexa 488 (ThermoFisher Scientific), at a 100‐fold dilution, were added in 2% BSA/PBS buffer and incubated for 2 h at RT in the dark. Sections were washed with 2% BSA/PBS buffer five times, then mounted in anti‐fade reagent Citifluor AF1 (Agar Scientific) containing 35 μg ml^−1^ of Calcofluor White M2R (Sigma‐Aldrich) for cell wall counter‐staining. Sections were observed with a confocal scanning microscope (Zeiss LSM700) and images were processed with imagej.

## AUTHOR CONTRIBUTIONS

TT designed and performed experiments and analysed the data. MB and FV performed some of the experiments. KS performed and analysed the NMR. RDM provided technical assistance to TT. TT and PD conceived the original research plans and wrote the article. PD agrees to serve as the author responsible for correspondence.

## CONFLICT OF INTEREST

The authors declare that they have no conflicts of interest associated with this work.

## Supporting information


**Figure S1.** Solution nuclear magnetic resonance (NMR) analysis.

**Figure S2.** MALDI‐LIFT MS/MS of the XA^3^XU^2^X oligosaccharide released by GH30 glucuronoxylanase from *Miscanthus* culms labelled with 2‐AA.

**Figure S3.** Schematic representation of the GH10 endoxylanase action on the xylan backbone.

**Figure S4.** HPAEC‐PAD analysis of SEC fraction 1 (SEC_fr1) and fraction 2 (SEC_fr2).

**Figure S5.** Indirect immunofluorescence detection of xylan epitopes in whole transverse sections of *Brachypodium distachyon* internodes before and after treatment of sections with GH30 glucuronidase or GH5 arabinoxylanase.

**Table S1.** ^1^H and ^13^C NMR assignments of all oligosaccharide structures in Figure S1, at 25°C in D_2_O.

## Data Availability

All relevant data can be found within the article and its supporting materials. To obtain raw data or materials, please contact the corresponding author.
