# Supplementary figures and images for: Grass xylan structural variation suggests functional specialization and distinctive interaction with cellulose and lignin
Source: Plant J. 2023 Jan 19;113(5):1004–20. doi: 10.1111/tpj.16096 (PMC10952629; doi:10.1111/tpj.16096)

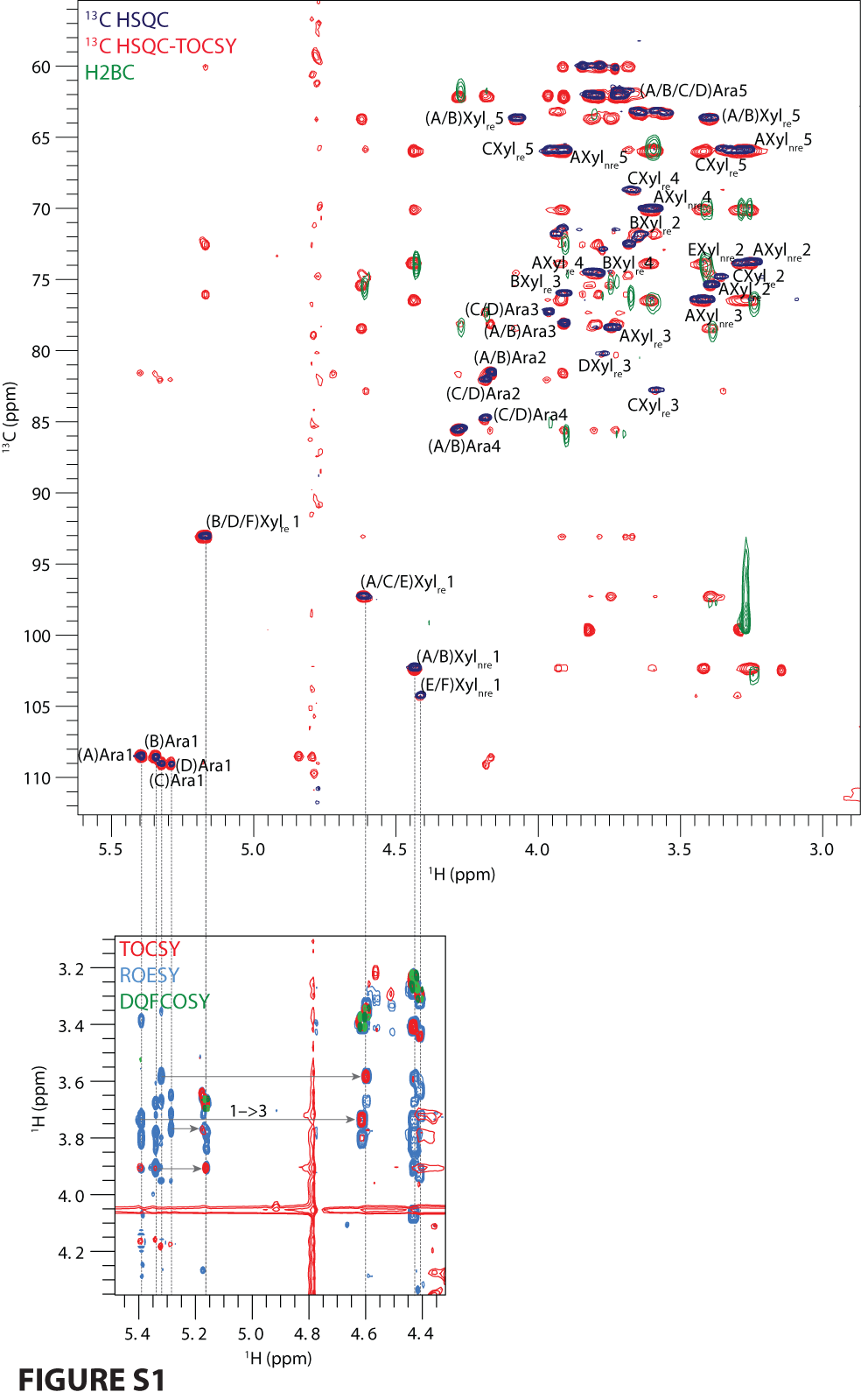

Supplement: Supplementary file 1 — Figure S1. Solution nuclear magnetic resonance (NMR) analysis. [file TPJ-113-1004-s004.png]

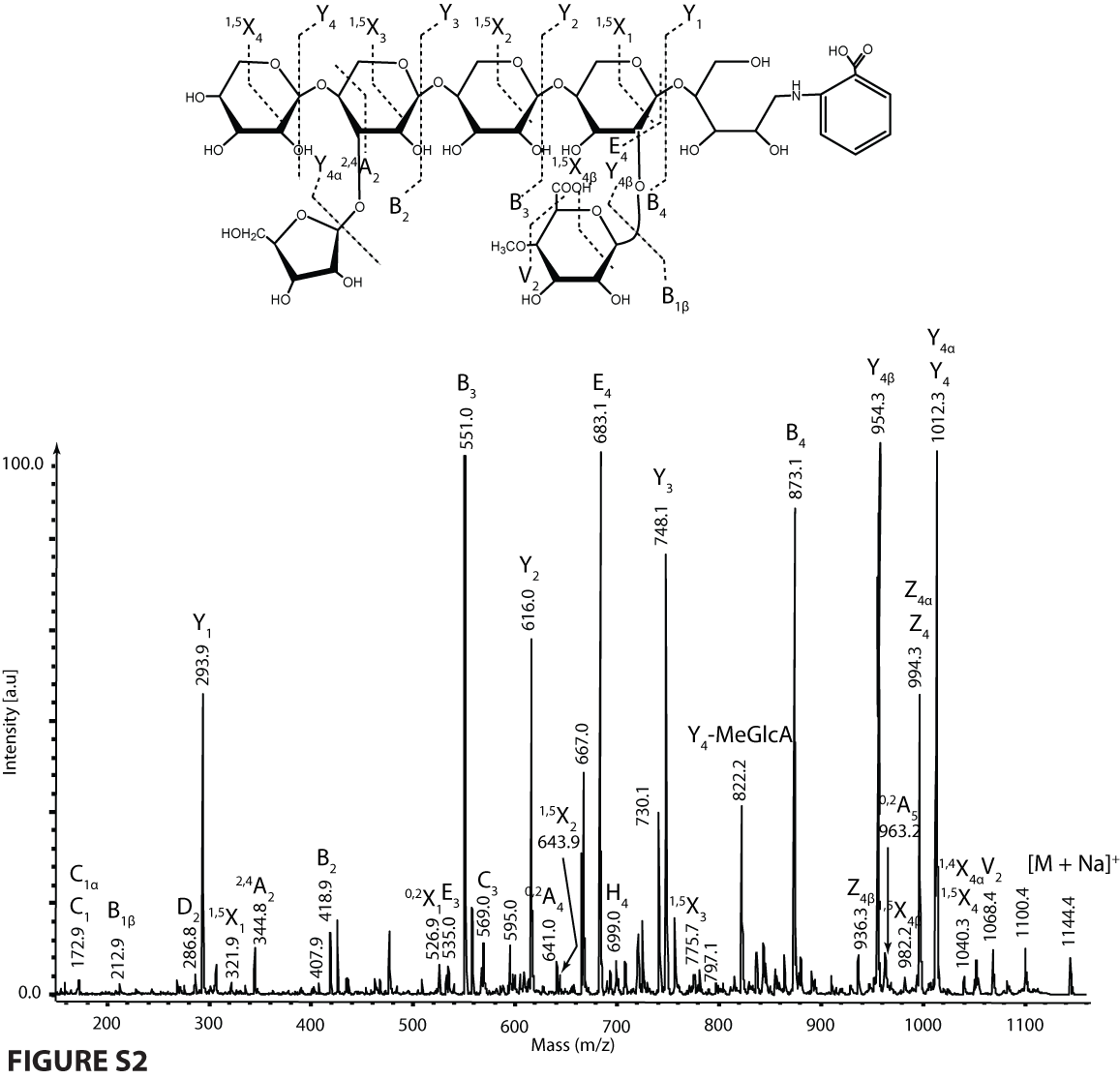

Supplement: Supplementary file 2 — Figure S2. MALDI‐LIFT MS/MS of the XA3XU2X oligosaccharide released by GH30 glucuronoxylanase from Miscanthus culms labelled with 2‐AA. [file TPJ-113-1004-s005.png]

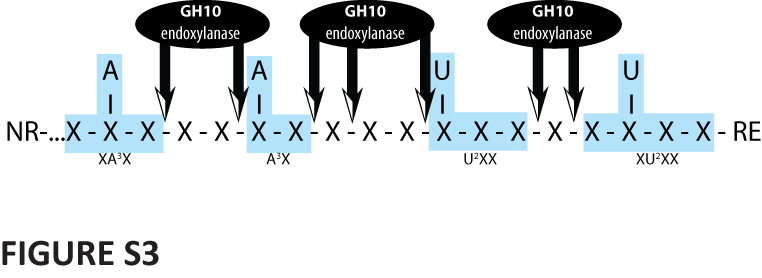

Supplement: Supplementary file 3 — Figure S3. Schematic representation of the GH10 endoxylanase action on the xylan backbone. [file TPJ-113-1004-s002.png]

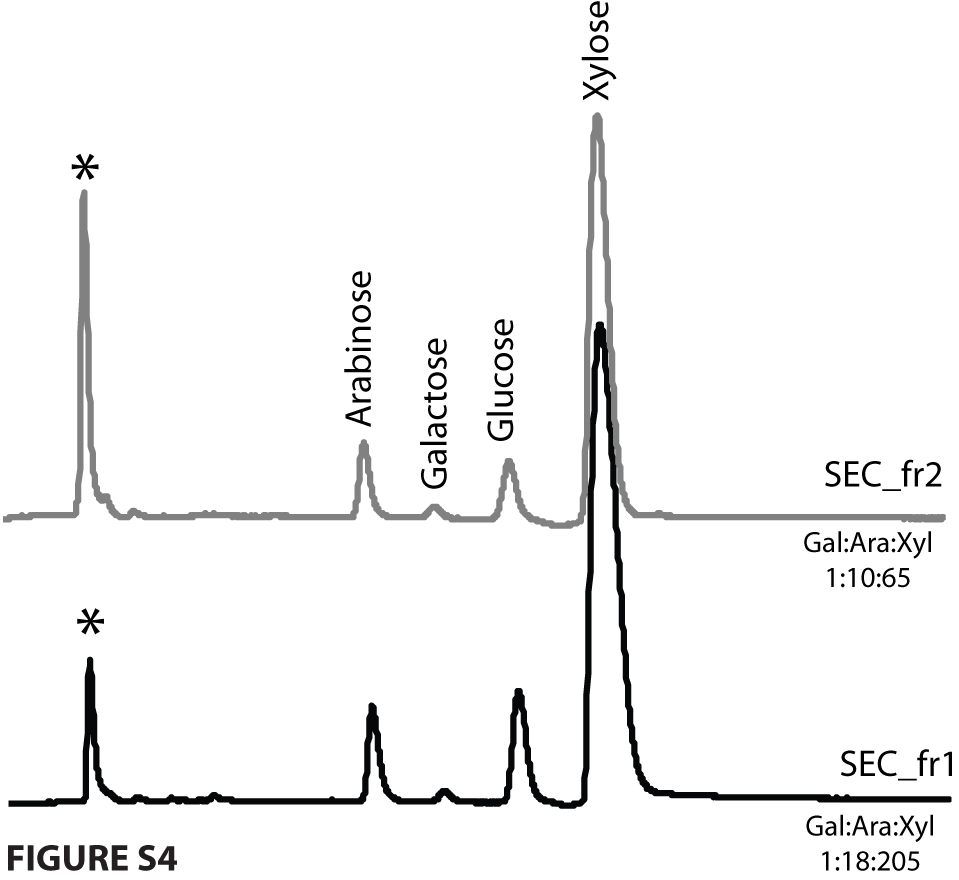

Supplement: Supplementary file 4 — Figure S4. HPAEC‐PAD analysis of SEC fraction 1 (SEC_fr1) and fraction 2 (SEC_fr2). [file TPJ-113-1004-s001.png]

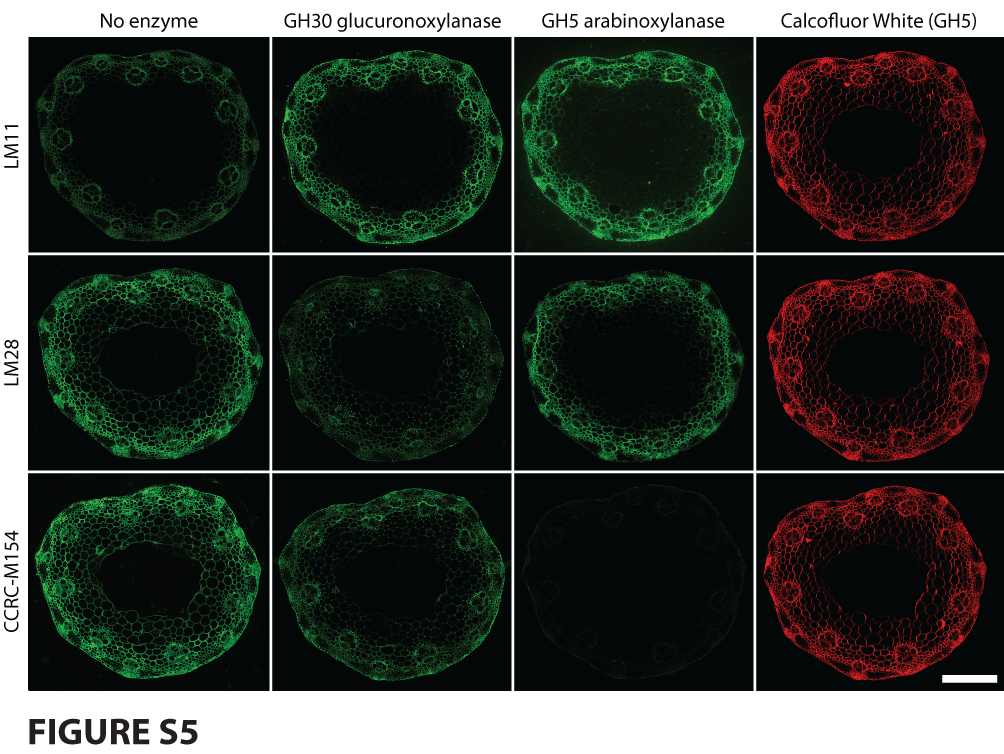

Supplement: Supplementary file 5 — Figure S5. Indirect immunofluorescence detection of xylan epitopes in whole transverse sections of Brachypodium distachyon internodes before and after treatment of sections with GH30 glucuronidase or GH5 arabinoxylanase. [file TPJ-113-1004-s003.png]
