# Supplementary material for: Grass xylan structural variation suggests functional specialization and distinctive interaction with cellulose and lignin
Source: Plant J. 2023 Jan 19;113(5):1004–20. doi: 10.1111/tpj.16096 (PMC10952629; doi:10.1111/tpj.16096)
Supplement: Supplementary file 6 — Table S1. 1H and 13C NMR assignments of all oligosaccharide structures in Figure S1, at 25°C in D2O. [file TPJ-113-1004-s006.docx]

**TABLE S1.** ^1^H and ^13^C NMR assignments of all oligosaccharide structures in Figure S1, at 25 °C in D_2_O.

|  |  | **Assignment** | | | | |  |  |
| --- | --- | --- | --- | --- | --- | --- | --- | --- |
| **Residue** |  | **1** | **2** | **3** | **4** | **5** | **Letter code** | **Chemical structure** |
| β-Xyl*p*_re_ | **^1^H** | 4.61148 | 3.38972 | 3.73816 | 3.80947 | 3.39795, 4.07237 | A | β-Xyl*p*-(1→4)-[α-Ara*f*-(1→3)]-**β-Xyl*p*** |
|  | **^13^C** | 97.24129 | 75.34189 | 78.35382 | 74.45799 | 63.6221, 63.61985 |  |  |
| α-Ara*f* | **^1^H** | 5.39459 | 4.1.1637 | 3.90588 | 4.27708 | 3.7261, 3.80306 | A | β-Xyl*p*-(1→4)-[**α-Ara*f***-(1→3**)**]-β-Xyl*p* |
|  | **^13^C** | 108.45818 | 81.49075 | 78.0336 | 85.50403 | 62.16128, 62.1662 |  |  |
| β-Xyl*p*_nre_ | **^1^H** | 4.4337 | 3.24666 | 3.41238 | 3.59728 | 3.27469, 3.91793 | A | **β-Xyl*p***-(1→4)-[α-Ara*f*-(1→3)]-β-Xyl*p* |
|  | **^13^C** | 102.25299 | 73.75139 | 76.37154 | 69.9905 | 65.87892, 65.88342 |  |  |
| α-Xyl*p*_re_ | **^1^H** | 5.16737 | 3.68091 | 3.90888 | 3.7863 | 3.39795, 4.07237 | B | β-Xyl*p*-(1→4)-[α-Ara*f*-(1→3)]-**α-Xyl*p*** |
|  | **^13^C** | 93.000973 | 72.46946 | 75.93935 | 74.51505 | 63.6221, 63.61985 |  |  |
| α-Ara*f* | **^1^H** | 5.34317 | 4.1.1637 | 3.90588 | 4.27708 | 3.7261, 3.80306 | B | β-Xyl*p*-(1→4)-[**α-Ara*f***-(1→3)]-α-Xyl*p* |
|  | **^13^C** | 108.54299 | 81.49075 | 78.0336 | 85.50403 | 62.16128, 62.1662 |  |  |
| β-Xyl*p*_nre_ | **^1^H** | 4.4337 | 3.24666 | 3.41238 | 3.59728 | 3.27469, 3.91793 | B | **β-Xyl*p***-(1→4)-[α-Ara*f*-(1→3)]-α-Xyl*p* |
|  | **^13^C** | 102.25299 | 73.75139 | 76.37154 | 69.9905 | 65.87892, 65.88342 |  |  |
| β-Xyl*p*_re_ | **^1^H** | 4.61148 | 3.35673 | 3.58401 | 3.67408 | 3.35094, 3.96305 | C | α-Ara*f*-(1→3)-**β-Xyl*p*** |
|  | **^13^C** | 97.24129 | 74.78376 | 82.75428 | 68.67639 | 65.80361, 65.87669 |  |  |
| α-Ara*f* | **^1^H** | 5.32408 | 4.17868 | 3.9606 | 4.18548 | 3.71163, 3.82857 | C | **α-Ara*f***-(1→3)-β-Xyl*p* |
|  | **^13^C** | 109.00215 | 81.95701 | 77.24486 | 84.71943 | 62.05628, 61.98726 |  |  |
| α-Xyl*p*_re_ | **^1^H** | 5.16737 | n.d | 3.77093 | n.d | n.d | D | α-Ara*f*-(1→3)-**α-Xyl*p*** |
|  | **^13^C** | 93.000973 | n.d | 80.20148 | n.d | n.d |  |  |
| α-Ara*f* | **^1^H** | 5.28935 | 4.17868 | 3.9606 | 4.18548 | 3.71163, 3.82857 | D | **α-Ara*f***-(1→3)-α-Xyl*p* |
|  | **^13^C** | 109.04328 | 81.95701 | 77.24486 | 84.71943 | 62.05628, 61.98726 |  |  |
| β-Xyl*p*_re_ | **^1^H** | 4.61148 | n.d | n.d | n.d | n.d | Ε | β-Xyl*p*-(1→4)-**β-Xyl*p*** |
|  | **^13^C** | 97.24129 | n.d | n.d | n.d | n.d |  |  |
| β-Xyl*p*_nre_ | **^1^H** | 4.41357 | 3.28999 | n.d | n.d | n.d | Ε | **β-Xyl*p***-(1→4)-β-Xyl*p* |
|  | **^13^C** | 104.23453 | 73.83201 | n.d | n.d | n.d |  |  |
| β-Xyl*p*_re_ | **^1^H** | 5.16737 | n.d | n.d | n.d | n.d | F | β-Xyl*p*-(1→4)-**α-Xyl*p*** |
|  | **^13^C** | 93.000973 | n.d | n.d | n.d | n.d |  |  |
| β-Xyl*p*_nre_ | **^1^H** | 4.41357 | n.d | n.d | n.d | n.d | F | **β-Xyl*p***-(1→4)-α-Xyl*p* |
|  | **^13^C** | 104.23453 | n.d | n.d | n.d | n.d |  |  |
